# Role of Molecular Water Layer State on Freezing Front Propagation Rate and Mode Studied with Thermal Imaging

**DOI:** 10.1021/acs.langmuir.4c00323

**Published:** 2024-06-14

**Authors:** Miisa J. Tavaststjerna, Stephen J. Picken, Santiago J. Garcia

**Affiliations:** † Department of Aerospace Structures and Materials, Faculty of Aerospace Engineering, Delft University of Technology, Kluyverweg 1, Delft, HS 2629, The Netherlands; ‡ Department of Chemical Engineering, Faculty of Applied Sciences, Delft University of Technology, Van der Maasweg 9, Delft, HZ 2629, The Netherlands

## Abstract

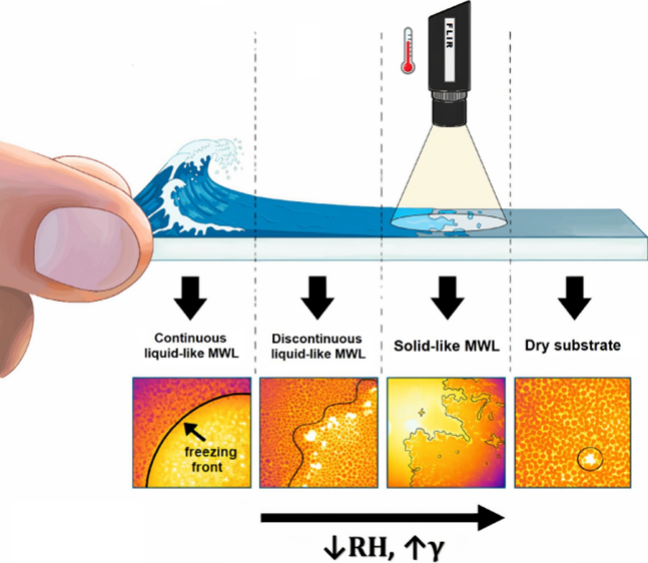

In this work, we study the relationship between the molecular water layer (MWL) and frost freezing onset and propagation. The progression of frost has been reported to be governed by various localized icing phenomena, including interdroplet ice bridging, dry zones, and frost halos. Reports studying the state of water on surfaces have revealed the presence of a thin nanometer water layer on a range of surfaces. Regardless of further investigations that show environmental humidity, temperature, and surface energy to affect the thickness of the MWL on surfaces, the influence of the MWL on frost nucleation and propagation has not yet been previously addressed in the literature. To study the effect of the MWL on surface freezing events, a range of surface-functionalized glass substrates were prepared. In situ monitoring of freezing events with thermal imaging allowed studying the effect of surface chemistry and environmental relative humidity (RH) on the thickness and continuity of the MWL. We argue that the observed icing nucleation and propagation kinetics are directly related to the presence and continuity of the MWL, which can be manipulated by controlling the environmental humidity and surface chemistry.

## 1 Introduction

The accumulation of ice on solid surfaces can have hazardous consequences, in applications as wide ranging as aircraft,^1,2^ power lines,^3^ marine vessels,^4^ microelectronics,^5^ and wind turbines.^6−8^ In the aerospace industry, icing affects negatively both the performance and safety of aircraft, whereas in the production of energy from the kinetic energy of air in motion, ice accumulation on the blades of wind turbines can significantly reduce their power generation efficiency. Ice accretion is currently managed by heating, using deicing fluids, and/or mechanically removing ice from the exposed surfaces. In spite of being efficient, in most cases, these methods are costly and demand intense labor, excessive amounts of energy, and time.^9−12^

During the past 70 years, researchers have been designing passive anti-icing coatings to overcome the above-mentioned challenges.^13,14^ Instead of relentlessly removing ice from a surface, an effective anti-icing coating aims at preventing icing accretion itself, in which case the problematic deicing methods would remain only as complementary ice removal systems.^15,16^ An ideal coating would be one to provide sufficient and long-lasting performance (i.e., low erosion and high UV resistance) with easy application and scalable production. It is calculated that even though an effective anti-icing coating might initially cost more in comparison to the use of deicing fluids, in the long term, a permanent coating would still reduce the overall costs, effort, and time spent on ice protection.^9,17^

There has already been a variety of attempts to create durable and effective anti-icing coatings.^17,18^ Despite these efforts, the incomplete in-depth understanding of the factors necessary to prevent or control ice formation on surfaces and the scatter of relevant information addressing the underlying mechanisms of ice accretion and propagation limit progress. For a more rational anti-icing coating design, it is beneficial to learn more about the factors affecting ice nucleation, propagation, and adhesion on surfaces. Of particular interest in understanding ice nucleation and propagation is the interaction of water molecules in the environment with the surface at the molecular level.

Past research on freezing propagation has mostly attributed freezing propagation to the formation of interdroplet ice bridges. In this work, we propose an alternative mechanism based on the presence of so-called molecular water layers (MWLs). Continuous layers of molecular water imaged at room temperature were first reported in the 1990s on hydrophilic mica surfaces using atomic force microscopy (AFM).^19^ In the early 2010s, while studying the nanoscale condensation of water droplets on COOH-modified hydrophilic silicon surfaces under ambient conditions, it was proposed that these nanoscale water droplets are interconnected through a, nonoptically detectable, thin liquid layer of water.^20^

Despite the initial reports confirming the presence of a MWL on hydrophilic surfaces, its role on ice nucleation and propagation has been widely overlooked in the field of anti-icing surfaces, while its presence may explain some of the observations reported in the literature. One plausible reason to overlook its role is the difficulty to detect MWLs under ambient conditions with the most popular and available characterization methods used in icing research, e.g., with optical microscopy. Nanoscale clusters of individual water molecules on solid surfaces have been otherwise systematically studied in ultrahigh vacuum conditions and at low temperatures using scanning tunneling microscopy (STM).^21^ However, in ambient conditions, molecular layers of water have only recently been studied on smooth silica surfaces using AFM,^19,20,22^ X-ray reflectometry,^20^ X-ray photoelectron spectroscopy,^23^ attenuated total reflection infrared spectroscopy (ATR-IR),^24^ and sum frequency generation spectroscopy (SFG).^25,26^

In agreement with previous studies, it can be proposed that MWLs between 0.2 and 6 nm may be present on smooth hydrophilic surfaces exposed to ambient conditions. Considering the experimental evidence, the proposed model system of water on surfaces suggests the presence of a homogeneous monolayer of solid hexagonally arranged water molecules followed by a transitional layer of restricted mobility and a sequence of disordered layers of liquid water molecules.^24^ Not many reports have been published on the thickness of each of these proposed water layers (solid-like or liquid-like) and the effect of environmental conditions (*T*, RH) and surface chemistry on such layers. Nevertheless, reports on cleaned smooth glass surfaces in maximum relative humidity (95–100%) at ambient temperature (22 °C) suggest that the solid-like MWL starts to transition into a liquid-like MWL when the solid-like MWL thickness is more than 3 monolayers of water (∼1 nm). At that point, the liquid-like MWL grows to a maximum reported thickness of 18 monolayers (∼6 nm).^23^

Uniformity of the MWL has attracted much less attention. On hydrophilic mica surfaces, the MWL appears as a uniform layer when the RH is above 40% or as a discontinuous film with dry holes and/or smaller individual islands of molecular water unevenly distributed on the surface when the RH is below 40%.^19,22^ These observations are compatible with other works, reporting MWLs to exist as a solid-like layer below 30% RH and as a liquid-like layer above 60% RH on hydrophilic silicon substrates.^20,24^ Although most reports did not report MWLs on hydrophobic surfaces at ambient conditions, MWLs of about 2 nm were reported for very hydrophobic surfaces such as halocarbon wax or Teflon at room temperature and 80% RH.^23^ This proves that water can adsorb or/and absorb on hydrophobic surfaces in high-humidity environments, especially when the surfaces have irregularities, defects, or high roughness. On hydrophilic surfaces at room temperature, the measured thickness of MWLs decreases with increasing water contact angle of the surfaces and with increasing temperature (e.g., 65 °C).^20^

In this work, we systematically study the effect of surface chemistry and relative humidity on the presence of molecular water layers (MWLs) and its related role on ice nucleation and propagation at subzero temperatures. To this aim, we used smooth and rough glass surfaces functionalized by using silane chemistry. Thermal imaging and image correlation protocols were used to monitor and quantify freezing events with high temporal and spatial resolution. The work unveils the role of MWLs in frost ice nucleation and propagation kinetics and their mode of propagation, through hydrophilic and hydrophobic surfaces, as a function of the ambient relative humidity. This confirms the likelihood of freezing events to occur on porous superhydrophobic surfaces.

## 2 Experimental Section

### 2.1 Materials

Standard microscope glass slides purchased from Carl Roth (corners cut, without frosted edge, 26 × 76 × 1 mm) were used as substrates across the study. The silanes 11-acetoxyundecyltrichlorosilane (95%), *n*-octyltrichlorosilane (97%), tridecafluoro-1,1,2,2-tetrahydrooctyltrichlorosilane (perfluorooctyltrichlorosilane, 97%) were purchased from ABCR, and heptadecafluoro-1,1,2,2-tetrahydrodecyltrichlorosilane (perfluoro-decyltrichlorosilane, 97%) was purchased from Gelest and used as received. All remaining solvents and chemicals used in this study were purchased from Sigma-Aldrich and used as received. Silica gel (3.5 mm bead size, for desiccation) was purchased from Sigma-Aldrich and heated in the oven at 130 °C for 4 h before use.

### 2.2 Surface Activation of the Glass Slides

Activation of the reactive hydroxyls and removal of any contaminants from the glass surface were done using a wet chemical method based on HCl and MeOH. In the activation procedure, the glass slides were first immersed in a solution containing a 1:1 volume ratio of MeOH:HCl for 30 min. In a subsequent step, the slides were rinsed with distilled H_2_O and dried under N_2_. The activated and dried slides were directly analyzed with water contact angle goniometry. Some of the prepared samples were further modified with functional silanes to control the surface water contact angle, as explained below.

### 2.3 Surface Silanization

Covalent attachment of functional silanes to the cleaned glass slides was carried out with a vapor deposition method in a vacuum chamber.^27^ After drying under nitrogen flow, two glass slides and three droplets (150 μL) of one silane were placed in separate dishes inside a custom-made sealed aluminum chamber connected to a vacuum pump. Vapor deposition of the silane onto the glass slides was carried out at low pressure (20 mbar) for 2 h at room temperature. Subsequently, the glass slides were removed from the chamber and used for contact angle measurements and icing tests. The procedure was repeated with four different silanes to obtain a representative range of hydrophilic to hydrophobic smooth surfaces.

### 2.4 Sol–Gel Surface Treatment

A sol–gel process leading to a porous-silica-based coating was used to develop a superhydrophobic surface on glass. To this aim, methyl trichlorosilane (0.2 mL) was deposited on the top of a glass surface and left to dry at room temperature while being covered with a Petri dish. After being dried, the sample was heated for 1 h at 100 °C. The procedure was repeated three times to obtain thicker layers.

### 2.5 Water Contact Angle Measurements

Water contact angles (WCAs) were determined right after the surface activation and silanization processes. The measurements were made using a KSV CAM 200 optical contact angle goniometer. Static, advancing, and receding water contact angles were recorded by using the sessile and needle-in-the-sessile-drop methods. All measurements were repeated three times for each sample. For advancing (A-WCA) and receding (R-WCA) angles, the initial volume of the drop (3 μL) was first increased with a pumping speed of 15 μL s^–1^ until a maximum droplet size of 15 μL. Then, the volume of the droplet was decreased from 15 μL back to 3 μL using the same pumping speed of 15 μL s^–1^. Drop shape analysis of the images was done based on the Young–Laplace equation. All WCA measurements were carried out at an ambient temperature of 21 ± 2 °C and relative humidity of 40% ± 5%.

### 2.6 Measuring and Quantifying Freezing Events with Thermal Imaging

Freezing events on the substrates were monitored by using a FLIR A655sc thermal camera with a close-up lens (1.5 magnifying factor and 25 μm lateral resolution). An emissivity value of 0.9 was used during the recordings to prevent reflections from influencing the results. To induce and control freezing, the samples were cooled on two Peltier elements set in parallel (40 × 40 mm each) to obtain a uniform temperature distribution. The Peltier elements were connected to a heat sink and a small fan for heat dissipation. Each thermal video recording started shortly before switching on the Peltier plates, where the glass samples were set to reach −20 °C at 15 °C min^–1^ and ended 1 min after the freezing event was observed to have propagated across the surface. Each measurement was repeated at least 3 times per sample. Two types of experiments were conducted: (i) monitoring frost formation in the absence of water droplets and (ii) monitoring the freezing of water droplets previously deposited on the sample surfaces. In the former, the surfaces were kept at −20 °C until a freezing event was observed. Image analysis was used to quantify freezing onset times and the kinetics of the freezing propagation front. The second set of experiments was carried out similarly but with a single 5 μL distilled water droplet placed on top of the sample before the Peltier plates were cooled down. Image analysis was used to quantify the freezing kinetics of the droplet and its surroundings. To study the effect of environmental humidity on the freezing events, the thermal camera and Peltier elements were placed inside a glovebox. The humidity inside was lowered to 25% RH with dried silica gel and increased to 50% RH and 70% RH by placing a beaker of CaCl_2_ solution to the closed environment.^28,29^ A humidity equilibrium inside the glovebox was reached within 3 days each time the humidity was adjusted. All the images were analyzed with the analysis program FLIR Research Studio and the image processing program ImageJ.

## 3 Results and Discussion

### 3.1 Water Contact Angles

**Figure 1:**
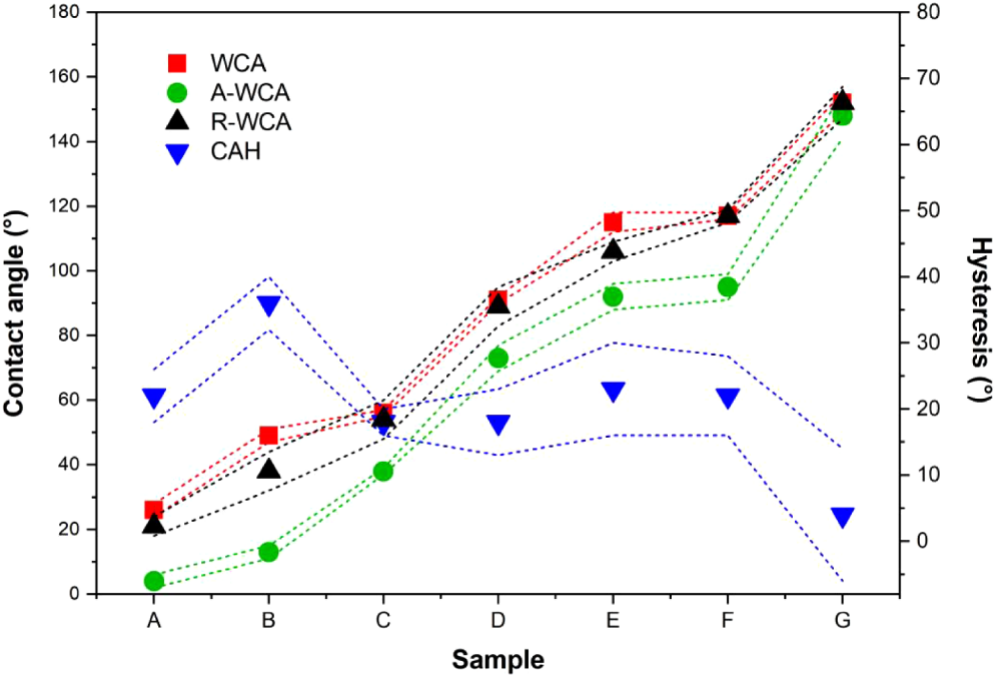
Water contact angles as a function of surface chemistry. The static (WCA), advancing (A-WCA), and receding (R-WCA) water contact angles of samples A–G show a similar increasing trend. The hysteresis (CAH) values are comparable for all smooth samples (A–F) and show a local minimum for the superhydrophobic sample (G).

**Figure 2:**
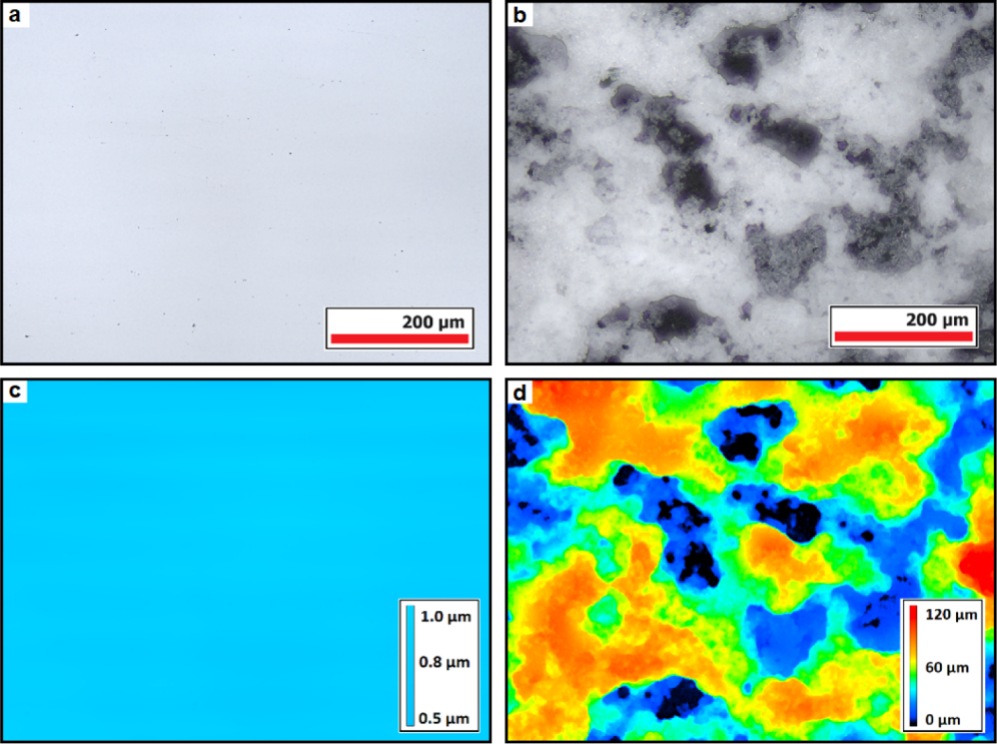
CLSM images show optical images (top row) and their corresponding height maps (bottom row) of the smooth glass surfaces functionalized with perfluorodecyltrichlorosilane (a,c) and the superhydrophobic surfaces prepared via sol–gel treatment (b, d).

Table 1 and Figure 1 summarize the measured static (WCA), advancing (A-WCA), and receding (R-WCA) water contact angles and hysteresis (CAH) as functions of the surface chemistry obtained after surface cleaning and silanization. As expected, the surface-activated glass slides show more hydrophilic surface chemistry (WCA = 21° ± 3°) than the degreased bare glass slides (WCA = 49° ± 2°) as a result of a larger presence of active hydroxyl groups.^30^ The WCA of the silane-treated samples follows the expected growing hydrophobic nature: acid end group < hydrocarbon chain < fluorocarbon content. Among the smooth surfaces, the glass slides covered with perfluorinated decyl carbon chains show the most hydrophobic WCA (109° ± 2°). Of all studied samples, the porous sol–gel coatings showed, as intended, superhydrophobic WCA (150° ± 5°). A-WCA and R-WCA followed a similar trend as WCA with surface chemistry. Interestingly, the surface activated and silanized smooth surfaces showed comparable CAH values (18–23°) lower than the CAH of the degreased bare glass surface (36° ± 4). The surface activation removes topological differences caused by contaminants, which can explain the lower CAH values on the surface-activated and silanized glass slides. The superhydrophobic surfaces, on the other hand, showed a significantly lower CAH (4° ± 10°); a distinctive mark of a superhydrophobic and self-cleaning surface as a result of roughness porosity entrapping air.^31^Figure 2 shows the confocal laser scanning microscopy (CLSM) images of the superhydrophobic surfaces and a smooth hydrophobic surface for comparison. The micrographs confirm the presence of porosity in the superhydrophobic surface responsible for air entrapment beneath water droplets. Altogether, these results confirm successful obtaining of a range of surfaces with varying hydrophilic/hydrophobic nature and justify the use of static contact angle (WCA) for the subsequent comparisons with the freezing events.

**Table 1:**
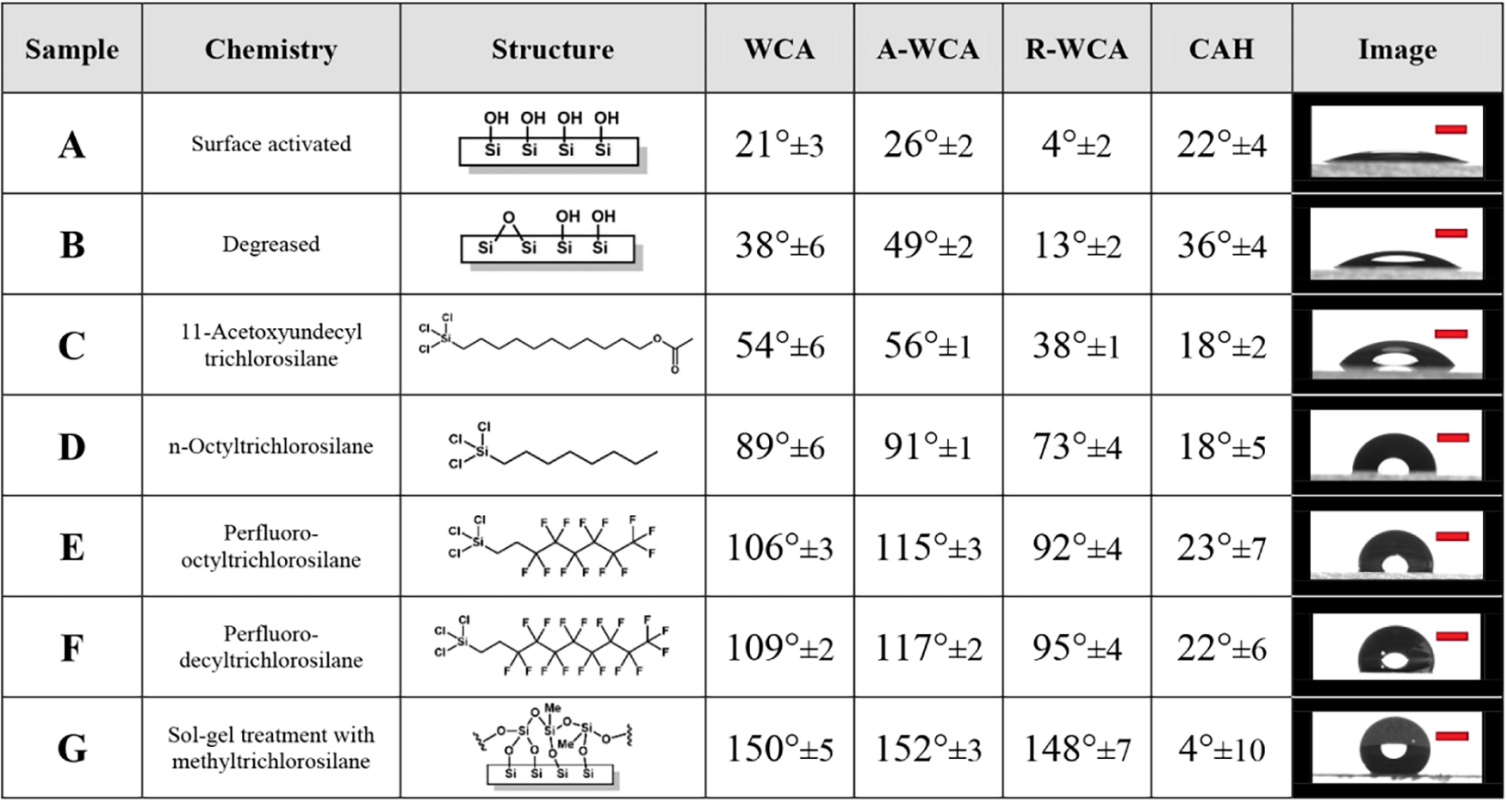
Series of Smooth and Rough Samples with Varying Surface Chemistry and Related WCAs^a^

a The red scale bar corresponds to 1 mm for all of the images shown here.

### 3.2 Frost Propagation in the Absence of Water Droplets

**Figure 3:**
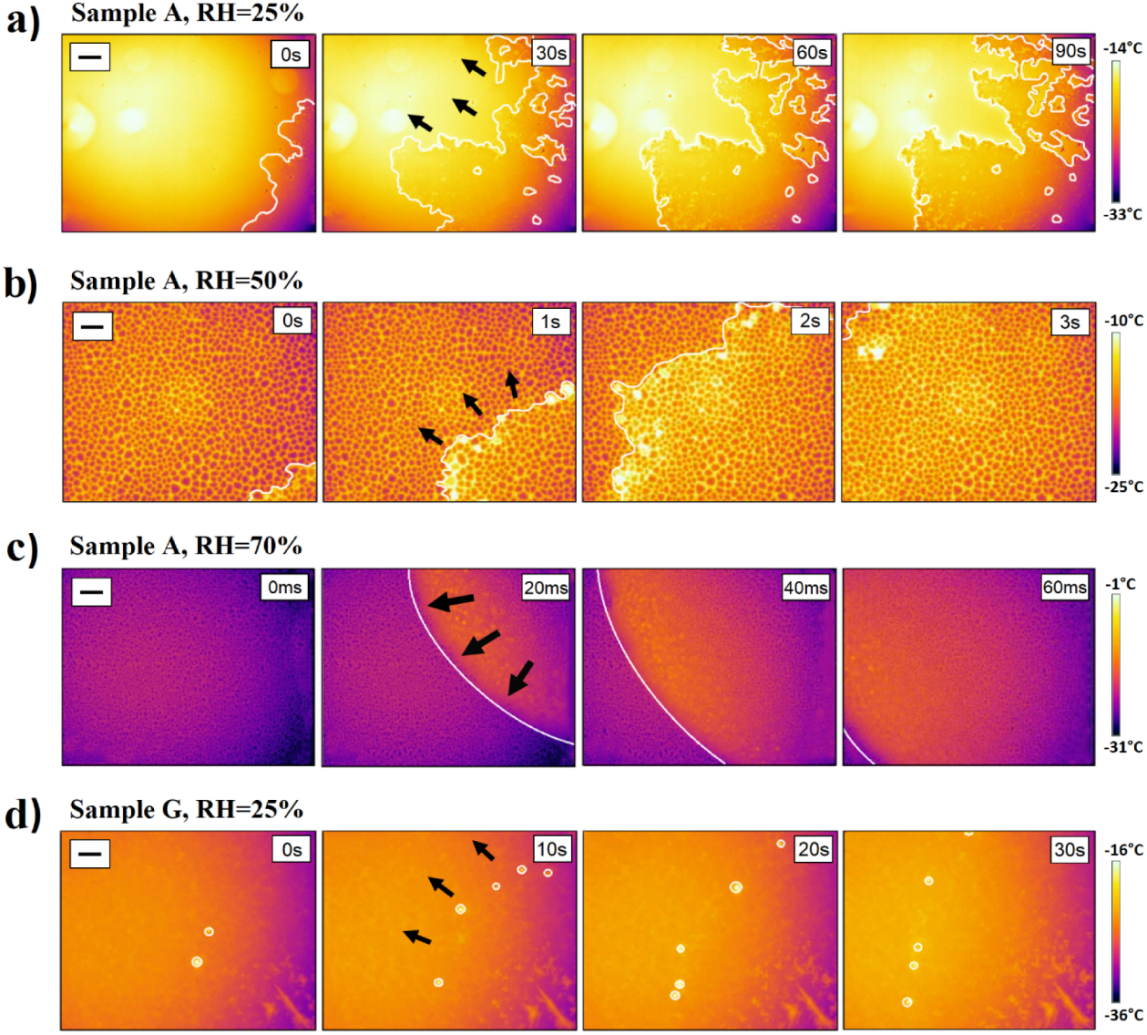
Time snapshots from thermal videos showing a freezing front propagating on the activated glass slides (sample A) as a function of relative humidity (a–c) and on superhydrophobic surfaces at 25% RH (d). The cooling rate in these experiments was set so that the sample surface reached −20 °C at 15 °C min^–1^. In a–c, the propagation front line is highlighted with a white line, and, in d, the isolated freezing events are marked with white circles. The black arrows indicate the direction of the freezing front propagation, and the black scale bars shown in the top left corners are 1 mm. The time indicated in the top right corner of each image shows the time step between each snapshot and, therefore, does not correspond to the freezing onset time. The vertical color bars indicate the temperature at the surface measured by the IR camera.

Figure 3 shows representative snapshots from the recorded thermal imaging videos as a function of the relative humidity (25%, 50%, and 70%) for surfaces (12 mm × 16 mm) without a droplet. The rest of the snapshots and videos can be found in Supporting Information (Tables S1–S3). In this experiment, thermal imaging reveals the latent heat release during the freezing of supercooled surface water. In practice, this allows following the freezing propagation front throughout the surface with high spatial and temporal resolution once a freezing event starts somewhere on the sample surfaces.

In the video snapshots, the dark purple color corresponds to a lower temperature and the bright yellow to a higher temperature. As liquid water freezes on the sample surface, a sudden temperature increase pinpoints the location of the freezing event. The higher the volume of water freezing, the more heat is released and the brighter the color change is in the video. In the case of a water droplet freezing, the abrupt temperature increase is eye-catching (see Section 3.3). On the other hand, it is much more difficult to follow the freezing front propagating on a surface with little or no water condensation present. Hence, in Figure 3a,b, the freezing front propagation fronts are highlighted with a white line, and the direction of the freezing front is specified with black arrows. After the latent heat has been released, the frozen areas keep cooling and appear with a darker color in the thermal videos.

At 25% RH, the propagation front line looks similar on all samples (Figure 3a and Table S1) except for the superhydrophobic surface (Figure 3d). There is no condensation visible on the surface (absence of small growing round dots with darker color compared to the substrate), and the freezing front line is clearly fractal-like in shape. In contrast, the superhydrophobic sample is covered with condensation, and as seen in Figure 3d, the freezing front line is only detectable by following individual condensed droplets lighting up as they freeze (marked with white circles).

At 50% and 70% RH, small droplets of condensation can be observed on all samples (Figure 3b,c and Tables S2 and S3). Instead of fractal-like freezing front lines, most of the samples show much smoother propagation fronts. The front line on the activated glass surface at 70% RH is almost perfectly round. Conversely, there were no changes in the freezing propagation pattern of the superhydrophobic surface, regardless of the humidity (Tables S1–S3), and the same freezing mechanism was observed on all hydrophobic silane-treated surfaces at 50% RH (Table S2).

### 3.3 Frost Propagation in the Presence of Deposited Water Microdroplets

**Figure 4:**
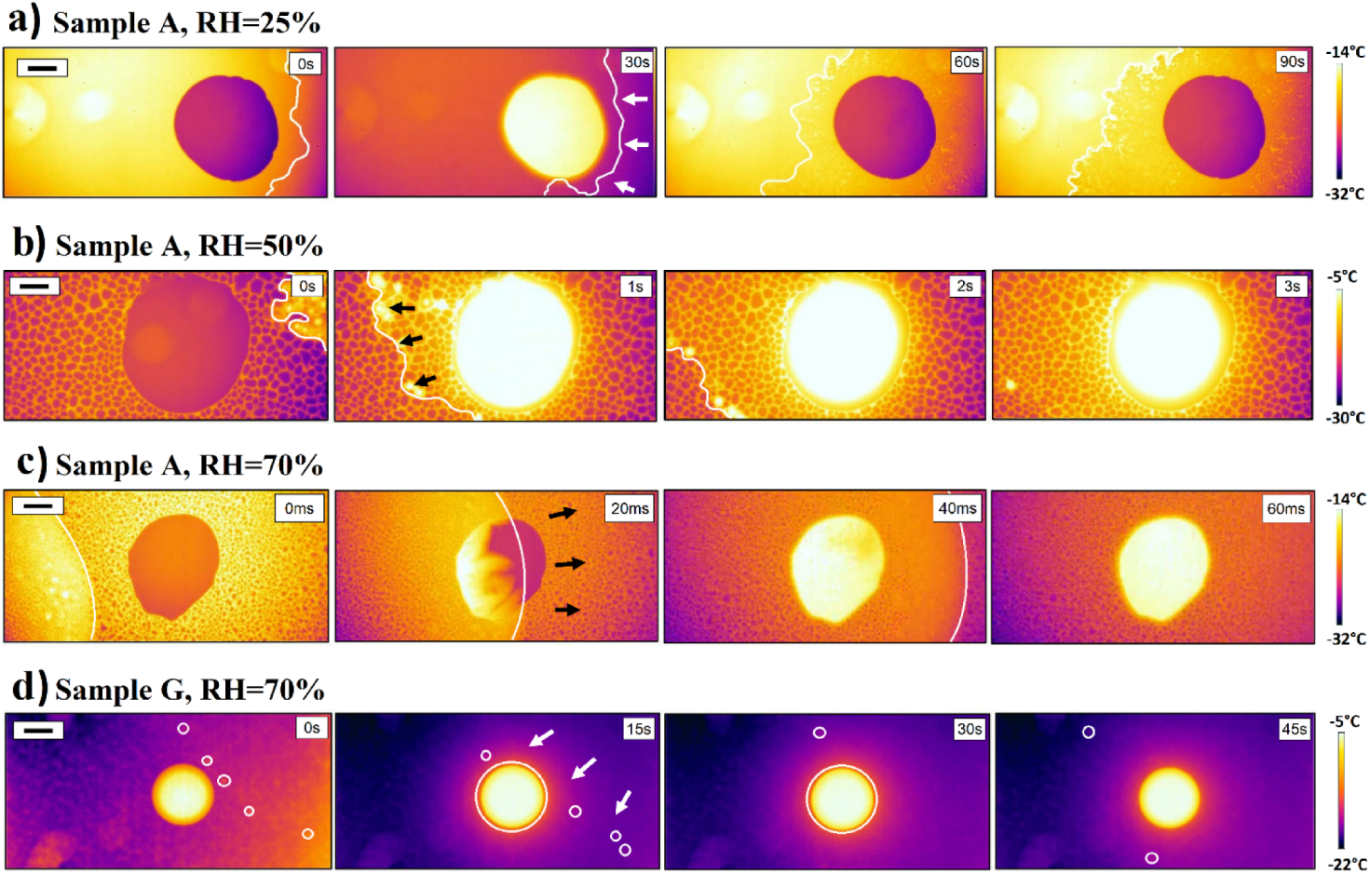
Time snapshots from thermal videos showing a freezing front propagating in the presence of a 5 μL water droplet on the activated glass slides as a function of relative humidity (a–c) and on superhydrophobic surfaces in 25% RH (d). The cooling rate in these experiments was set so that the sample surface reaches −20 °C at 15 °C min^–1^. In a to c, the front line of each freezing event is highlighted with a white line, and, in d, the isolated freezing events are marked by white circles. The white and black arrows indicate the direction of the freezing front propagation. The time indicated in the top right corner of each image shows the time step between each snapshot and, therefore, does not correspond to the freezing onset time. The black scale bars shown in the top left corners are 1 mm. The vertical color bars indicate the temperature at the surface measured by an IR camera.

Figure 4 shows representative snapshots from the recorded thermal imaging videos as a function of the relative humidity (25%, 50%, and 70%) in the presence of a water droplet (5 μL) deposited in the center of each sample. The complete set of samples is listed in Tables S4–S6. The main distinctive feature of these image snapshots is the presence of a large round supercooled droplet in the center lighting up when freezing. Notably, the droplet begins freezing only when the propagation front line meets it, as clearly seen in Figure 4c at the 20 ms snapshot. The presence of the droplet seems to not have influenced the freezing propagation modes discussed in Section 3.2.

### 3.4 Freezing of the Microdroplets

**Figure 5:**
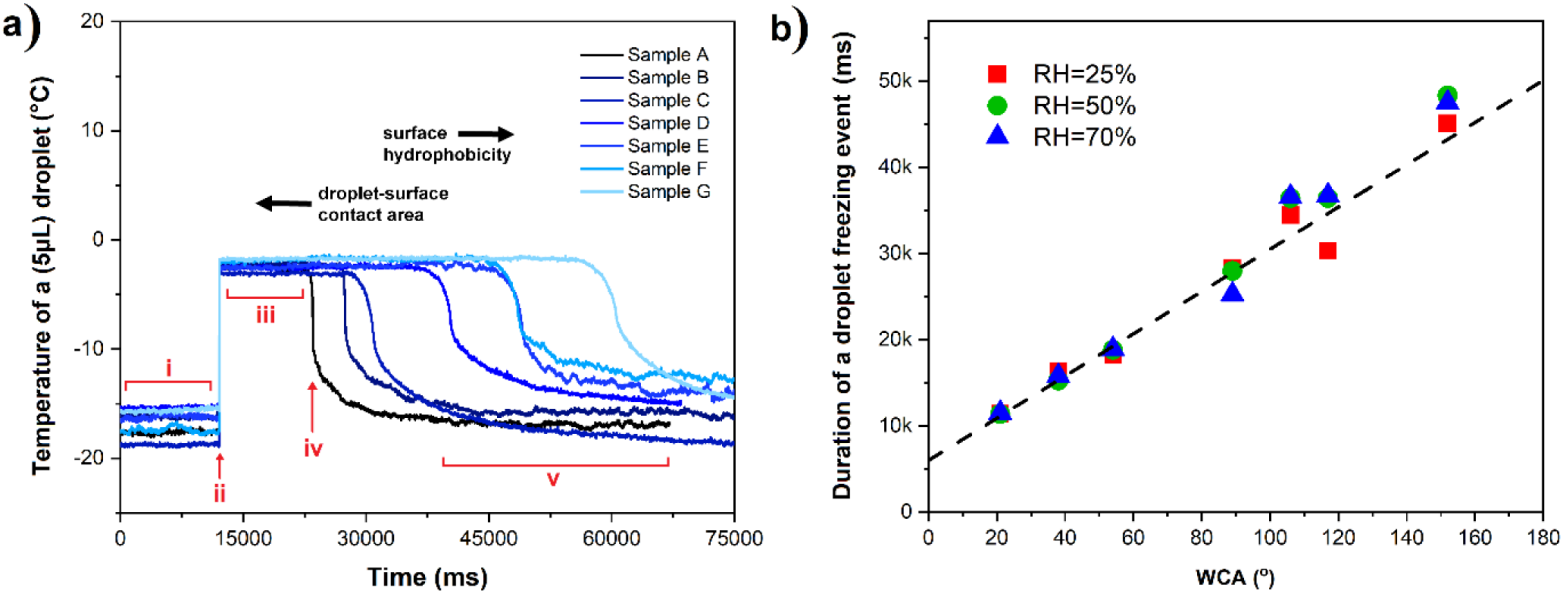
(a) Time–temperature plots from a 5 μL droplet freezing at 50% relative humidity. For sample A, the different stages of freezing are marked with red numerals i, ii, iii, iv, and v. The plots are set to overlap each other at *t*_onset_ (ii) to demonstrate the increase in freezing duration time with increasing hydrophobicity of the samples. (b) Duration of the freezing event (*t*_end_ – *t*_onset_) plotted as a function of the static WCA in 3 different humidities. A linear dependence between droplet freezing and static WCA is observed.

The high accuracy of thermal imaging allows extracting local temperature at any location and time, for instance, at a water droplet present at the surface as shown in Figure 4. Figure 5a plots the temperature of supercooled droplets on the different studied samples as a function of the freezing experiment time. Independently of the surface chemistry, all droplets show the same temperature profile during freezing: (i) supercooling to the temperature around −15 °C; (ii) sudden temperature increase to around −5 °C (*t*_onset_); (iii) isothermal at that temperature for varying times; (iv) rapid temperature drop (*t*_end_), and (v) stabilization to the initial temperature before the freezing event.

The sudden temperature increase (ii) originates from the latent heat released in the phase change from liquid to solid. Some of this heat release is observed as a temperature increase in the videos, yet a part of it is dissipated into the substrate. Figure 5b shows the extracted duration of the freezing event of individual droplets (*t*_onset_ until *t*_end_) as a function of the WCA. Even though the water droplets have the same volume in all of the experiments, the plot reveals a linear increase of the freezing event duration (length of stage (iii)) with the WCA. In general, the higher the contact area (the lower the contact angle), the earlier the freezing event ends (shorter stage (iii)). The superhydrophobic sample keeps the general trend and shows the longest freezing event duration, presumably due to having the lowest contact area and, therefore, slowest latent heat dissipation into the substrate and surrounding environment.

### 3.5 Freezing Onset Time at the Surface with and without a Droplet

**Figure 6:**
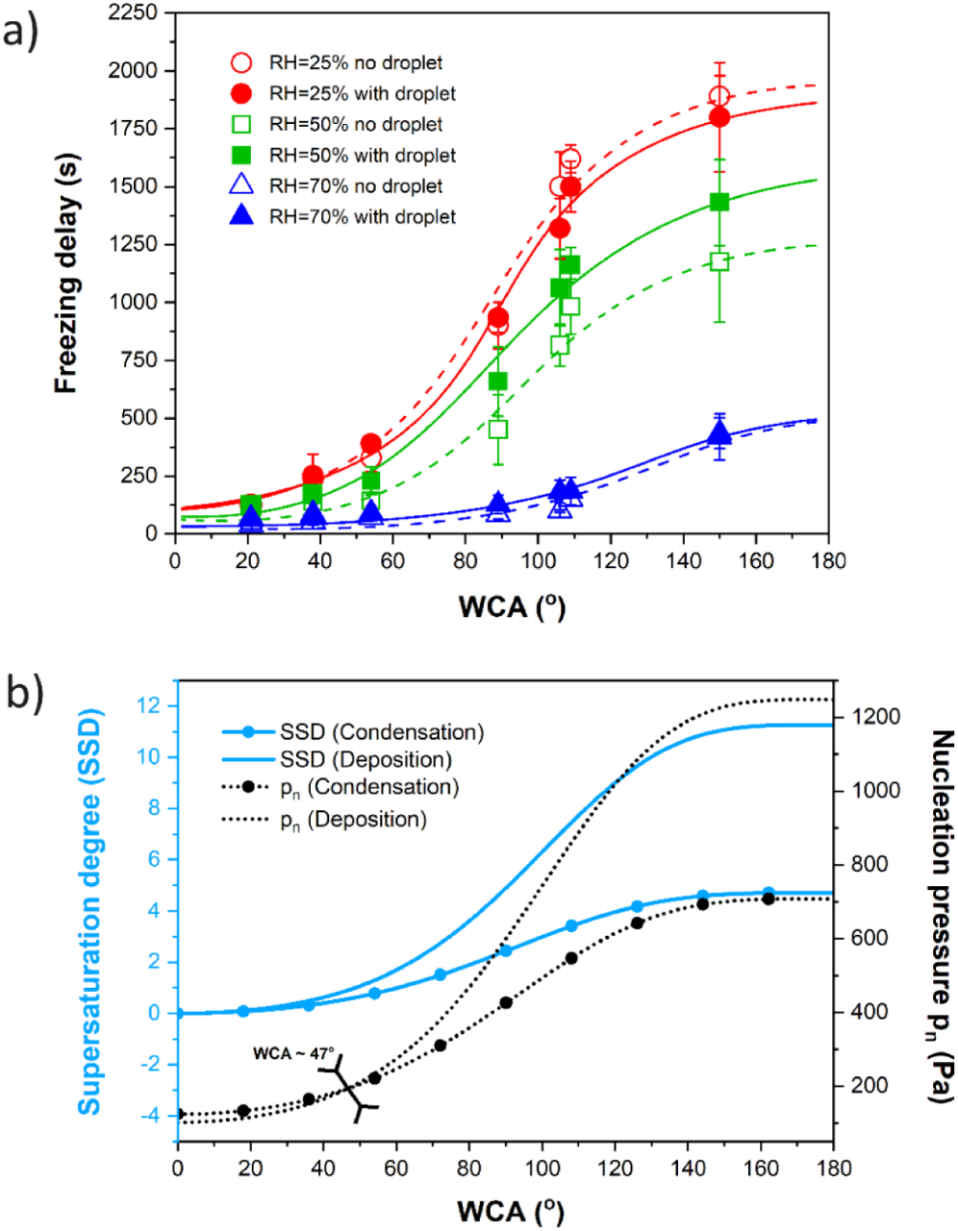
(a) Freezing onset times as a function of the static WCA of the sample surfaces in the experiments conducted with a 5 μL water droplet placed in the center of the sample (filled symbols, solid line) and without a water droplet (empty symbols, dashed line) in three RHs. The continuous and dashed lines serve as a guide to the eye. (b) Supersaturation degree (SSD) and nucleation pressure (*p*_n_), respectively, plotted as a function of the static WCA for condensation (vapor-to-liquid, marked with circle symbols) and deposition (vapor-to-solid, no symbols). The functions are calculated for −20 °C surface temperature using 10^29^ m^–2^ s^–1^ as a fixed kinetic constant for embryo formation for both condensation and deposition using eqs S1–S6 . The black inverted arrows indicate the WCA intersection point of nucleation pressure curves below which deposition is thermodynamically favored (WCA < 47°).

From each freezing experiment recorded using the thermal camera (Figures 3, 4 and Tables S1–S6), a freezing onset time (*t*_fo_) was extracted and plotted in Figure 6a as a function of the surface chemistry (represented by the WCA) and the relative humidity (RH). The freezing onset time is described as the time from when the cooling plates are turned on to the moment at which the first freezing event is captured by the observation window of the thermal camera.

In Figure 6a, the freezing experiments done with and without a droplet show the same trend shape for every relative humidity. The freezing onset times grow exponentially with increasing WCA until reaching an apparent plateau at WCA > 120° with the superhydrophobic samples, showing much lower freezing onset times than expected. However, without additional data points at WCA > 150°, it is not possible to confirm what happens to the trend in the case of superhydrophobic surfaces.

Similarly, freezing onset times also decrease as the RH increases. The presence of the droplet did not seem to have a large effect on the results at RH 25% and 70% nor on the freezing onset values of any of the hydrophilic surfaces at any RH. In the case of RH 50%, the presence of the droplet seems to have delayed the freezing onset times of the hydrophobic samples; however, all freezing onset measurements at RH 50% fit between rather wide error bars.

A similar exponential growth toward a plateau has been found for the dependence of the supersaturation degree (SSD) and nucleation pressure on WCA, following the mathematical procedure by Nath and Boreyko^32^ (eqs S1–S6). In the experiments therein, there are two possible mechanisms that explain frost development on the sample surfaces: (i) water first condenses on the sample surfaces and then freezes via condensation frosting or (ii) water deposits directly from vapor to solid ice. The phase change of water vapor to liquid (condensation) or solid ice (deposition) on a substrate requires either an undercooling of the substrate temperature or supersaturation of the surrounding vapor pressure. SSD is a parameter that describes the extent of supersaturation needed for condensation or deposition to occur on the substrate at a given temperature, whereas the nucleation pressure gives the pressure required for each phase change. The values of SSD and nucleation pressure are both dependent on substrate wettability and therefore have different values for each of the samples.

In Figure 6b, the SSD and nucleation pressure have been plotted as a function of WCA for both condensation and deposition at −20 °C. The similarities between plots 6a and 6b can be understood, since both SSD and freezing onset times can be used to describe the energy barrier of nucleation on surfaces. It is also notable that, in Figure 6b, the nucleation pressure required for deposition becomes lower than the nucleation pressure required for condensation at WCA < 47°, indicating that deposition is theoretically a more favorable mode of nucleation on the hydrophilic surfaces at −20 °C.

### 3.6 Relation between Freezing Front Propagation, Surface Chemistry, and MWL

**Figure 7:**
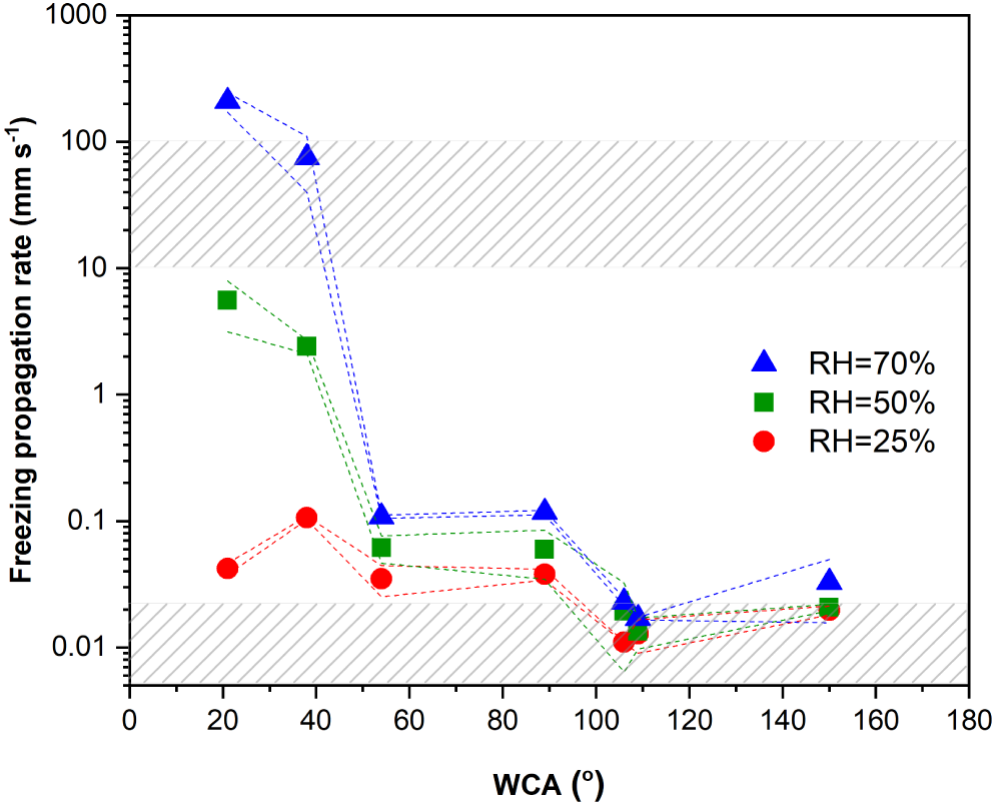
Freezing front propagation rates (mm s^–1^) as a function of the static WCA measured at three RHs in the absence of a droplet. The error bars of the freezing front propagation rates are indicated by the dashed lines for each RH. The upper and bottom gray pattern blocks mark the range of propagation rates previously reported for intradroplet freezing (10–100 mm s^–1^) and percolation-induced frost growth (∼0.01 mm s^–1^), respectively.

Previous research dedicated to freezing propagation phenomena on surfaces mostly focused on frost growth using optical microscopy under experimental conditions leading to visible water droplet condensation.^33−44^ Under these circumstances, individual droplets sequentially freeze when optically detectable ice bridges, formed at a frozen droplet, propagate and reach a neighboring liquid droplet. In line with these observations, freezing propagation on surfaces has been related to the formation of interdroplet ice bridges (i.e., percolation-induced frost propagation). Experimental evidence^35−41^ suggests that the propagation rate of such interdroplet bridges occurs at ∼0.01 mm s^–1^, 4 to 5 orders of magnitude slower than the reported intradroplet freezing propagation rate (10–100 mm s^–1^).^45−48^ As a consequence, the overall frost propagation rate on surfaces has been reported to be around 0.01 mm s^–1^ based on a (relatively) limited number of substrates and environmental conditions studied. In this work, we identified different freezing front propagation mechanisms and rates as a function of the relative humidity, substrate hydrophilicity, and topology. Figures 3 and 4 and Tables S1–S6 show snapshots of the freezing experiments monitored by thermal imaging used in this work to calculate freezing front propagation rates and to identify freezing propagation mechanisms as a function of RH and surface energy. Analysis of the images and identification of the freezing front propagation lines were performed using the image processing program ImageJ. This allowed us to identify different freezing front lines depending on the experimental conditions (front lines marked as white lines in Figures 3 and 4 and Tables S1–S6). Replicates of the freezing experiments allowed obtaining average freezing propagation rates with deviation for each experimental (RH) and sample surface energy (WCA) as shown in Figures 7 and Figure S3.

Figure 7 shows how, independent of the front-line geometry and propagation mechanism, the freezing fronts propagate faster at higher environmental humidities. Larger differences are nevertheless seen at low surface energies (WCA in the range of 20–60°) and high relative humidities (50% and 70%). This trend seems to hold true for all of the experimental conditions except for the superhydrophobic surfaces (WCA ∼ 150°) for which the freezing front propagation rates increase at all RHs slightly above the most hydrophobic of all smooth surfaces (both perfluorinated samples with WCA around 105–110°). The increase in the freezing propagation rate for the superhydrophobic sample is attributed here to moisture entrapment within the porous structure of the superhydrophobic samples shown in Figure 2.

In addition to the values of propagation rates, valuable information about the freezing propagation mechanisms can be obtained by visual examination of the thermal imaging videos. As seen in Figures 3 and 4 and Tables S1–S6, the shape of the freezing front can vary dramatically from a smooth round line (e.g., sample A with WCA ∼ 21° at 70% RH) to a complicated fractal border (e.g., sample C with WCA ∼ 54° at 25% RH). When examining simultaneously the propagation rates and the shape of the propagation front line, it appears that the smoothness of the freezing front is directly related to faster freezing front propagation rates. Droplet condensation density, on the other hand, did not have a measurable effect on the mode and kinetics of ice propagation. When analyzing the freezing propagation fronts under all conditions (WCA and RH), four freezing front propagation modes can be identified. Table 2 summarizes the characteristics of these propagation modes and the conditions under which they were observed.

**Table 2:**
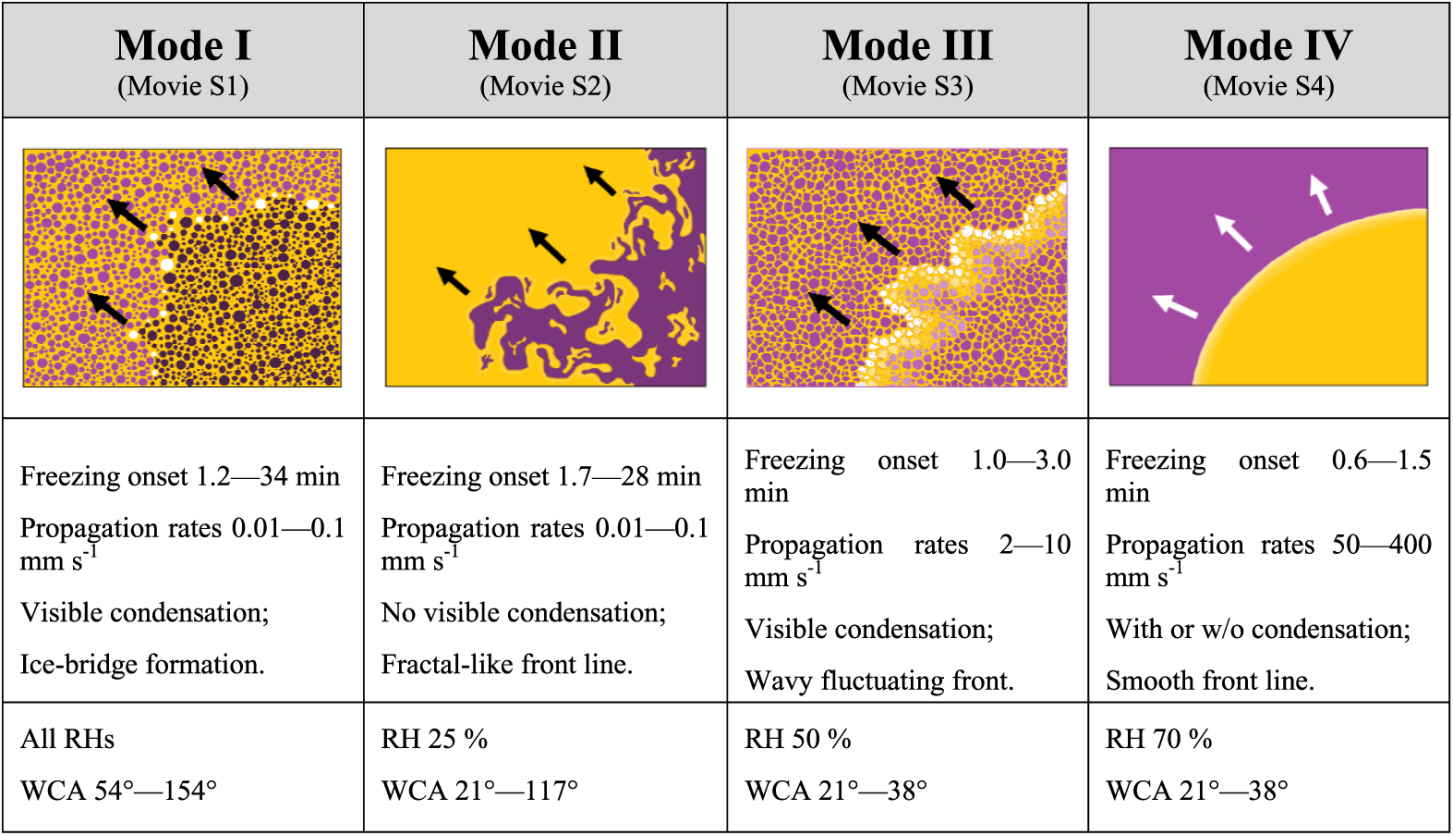
Characteristics of the Four Freezing Propagation Modes Observed via Thermal Imaging and the Conditions under Which They Are Observed

The specific freezing front propagation rates for all RHs and WCAs and their relation to the freezing propagation mode are shown in Table 3.

**Table 3:**
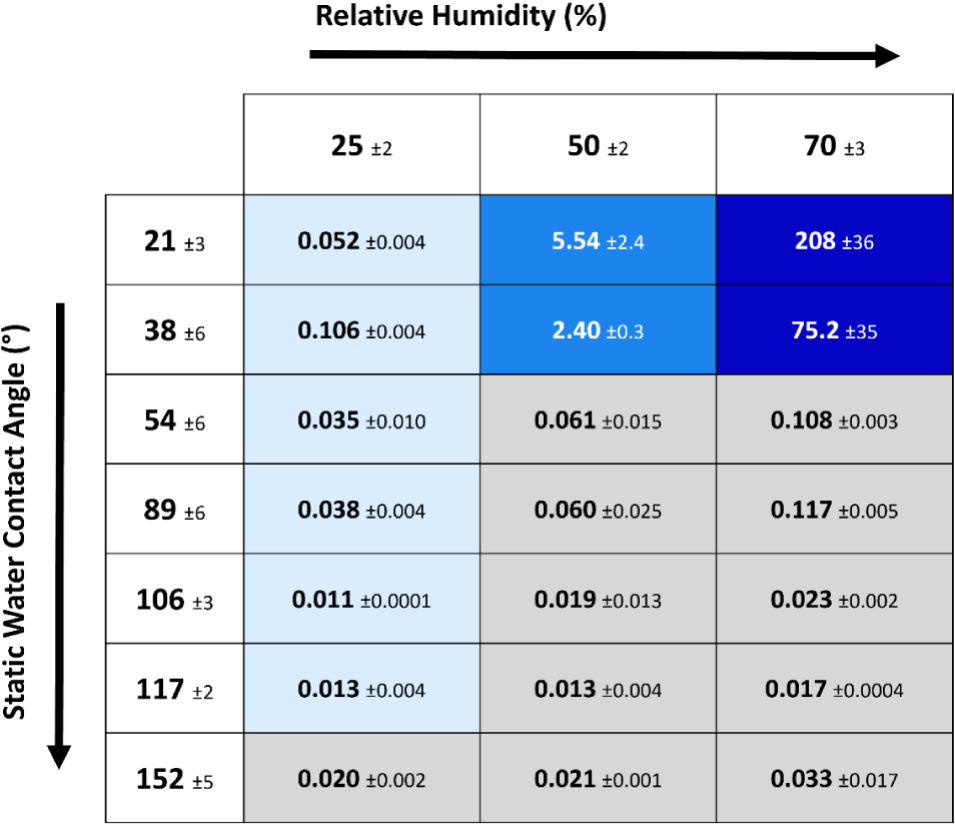
Quantified Freezing Propagation Rates (mm s^–1^) in Relation to RH and Surface WCA and Their Relation to the Freezing Propagation Mode^a^

a Gray background marks propagation mode I, light blue mode II, medium blue mode III, and dark blue mode IV.

As summarized in Tables 2 and 3, freezing propagation mode I and mode II show propagation rates in the same ballpark (0.01–0.1 mm s^–1^). The lower limit of this range (0.01 mm s^–1^) is well aligned with values previously reported for percolation-induced freezing as shown in Figure 7. However, the upper limit (0.1 mm s^–1^) observed in samples with both propagation modes is about 1 order of magnitude faster than percolated-induced freezing. This suggests the influence of another surface factor appearing at a certain RH and surface hydrophilicity that homogeneously affects the surface without visibly detectable water condensation. As summarized in Table 2, mode II (low-speed fractal propagation) appears to be limited to low relative humidity (25% RH) and smooth surfaces (independently of their hydrophilicity). The fractal-like propagation observed for mode II is well aligned with previous studies at low humidity (RH ≤ 35%) on hydrophobic PMMA and hydrophilic metallic substrates attributed to a visibly detectable deposition-limited frost growth.^49,50^

**Figure 8:**
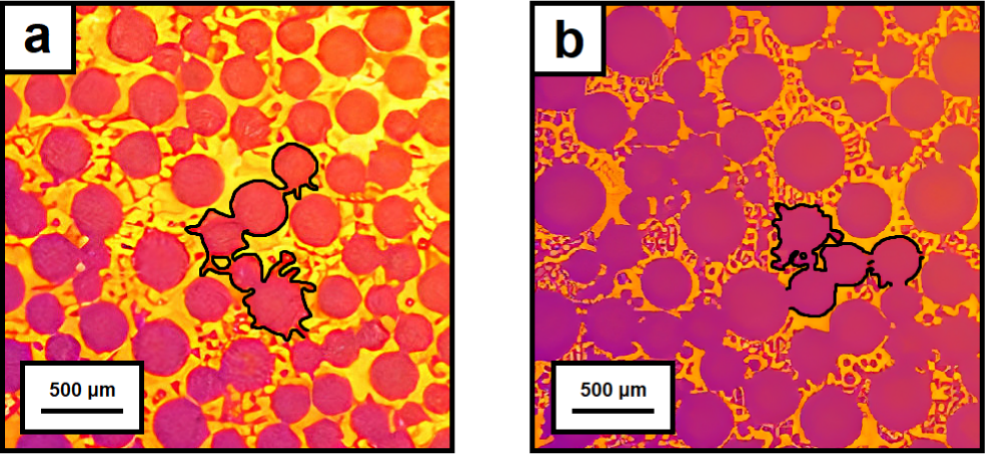
Time snapshot from a thermal imaging video showing the surface freezing of (a) sample E (WCA = 106°) at 50% RH and (b) sample D (WCA = 90°) at 70% RH with frozen condensation on top. The numerous connecting pathways between the frozen droplets (highlighted with a black line) are here identified as interdroplet ice bridges.

A detailed look at the thermal imaging video recordings (Tables S2, S3, S5, and S6) reveals the presence of small ice bridges in all the samples showing mode I propagation (e.g., see snapshots in Figure 8). The ice bridges’ presence is in good agreement, together with the average propagation rates around 0.01–0.1 mm s^–1^, with ice-bridge controlled frost propagation observed in samples freezing in the presence of condensation droplets.

The identification of these two propagation modes (mode I corresponding to ice-bridge-limited frost growth and mode II corresponding to deposition-limited frost growth) at all RHs and most surface energies (WCAs) hints at a relation between the propagation mode and the presence of water at the surface. In line with this, higher amounts of water at the surface should lead to faster and more homogeneous propagation front lines, as hereon discussed.

Frost propagation modes III and IV show significantly earlier freezing onset times (1–3 min) and faster propagation rates (2–400 mm s^–1^) than modes I and II, and more continuous propagation fronts instead of fractal lines. These two modes (III and IV) are observed only for the most hydrophilic samples (samples A and B with WCAs below 40°) at the highest RHs (50% and 70%). In general, a clear trend from a fractal frontline with a slow propagation rate to a continuous frontline with a fast propagation rate is observed when the RH and the hydrophilic nature of the surface increase. It is remarkable to note that the flash-like (see Movie S4) freezing propagation rates observed in mode IV for high RH and hydrophilic surfaces are 1 order of magnitude faster (up to 400 mm s^–1^) than the reported values of bulk water freezing (50 mm s^−151^) and several orders of magnitude slower than the speed of sound in water (1000 m s^−152^). The freezing propagation rate is similar to the speed of rapidly released vapor bolus (500 mm s^–1^) observed during cascade freezing in reduced pressure (∼3 mbar) reported in literature.^53^ In our work, the freezing experiments were conducted in normal atmospheric pressure, which would reduce the diffusion speed due to cascade freezing to a maximum of 3 mm s^–1^. These factors and the observation of the freezing front in the presence of nonfrozen individual water droplets from condensation (see Tables S2 and S5) rule out freezing propagation dominated by regular water freezing of a thick continuous water film layer created by condensation as well as the acceleration of the freezing propagation by an acoustic wave traveling in water or a vapor bolus released during droplet freezing cascade. However, comparable freezing propagation rates (200–400 mm s^–1^) have been reported for the intradroplet freezing of impacting supercooled water droplets on surfaces.^47,48^ Moreover, the experimental values obtained by us and those reported for supercooled droplets freezing are comparable and just 1 order of magnitude lower than those calculated with numerical simulations for freezing of supercooled water on ice (1000–10000 mm s^–1^ at RH 100% and WCA ∼ 0°).^54^

Altogether, the above considerations suggest freezing in modes III and IV took place through a supercooled water layer and pave the way to the relation between surface freezing and the presence of molecular water layers (MWL), whose thickness and continuity affect the freezing front propagation mode and rate. Even though we did not measure the thickness of the water layers for the samples showing mode III and IV propagation (i.e., hydrophilic surfaces, samples A and B, at high relative humidities (RH 50–70%)), a MWL of around 1 nm can be assumed based on previous experimental studies^19−26^ reporting MWLs of more than 1 nm thick on hydrophilic silicon surfaces at RH 60%. We hypothesize that, since thickness decrease accelerates freezing propagation rates, propagation delays observed in mode III compared to conditions leading to mode IV cannot be explained by the lower thickness induced by lower RH but by discontinuities in the MWL causing a wavy fluctuating propagation front line, with overall propagation rate decrease due to local differences in freezing propagation rates.

In line with the above, and as reported by others in the case of hydrophilic and hydrophobic chemically modified silicon wafers,^20^ there should be no liquid-like molecular water present on the sample surfaces in the case of modes I and II. However, there is 1 order of magnitude difference in the propagation kinetics of modes I and II (between 0.1 and 0.01 mm s^–1^). Based on literature, the highest propagation rates measured in conditions leading to propagation mode I and mode II (0.1 mm s^–1^) can be explained by the presence of a solid-like MWL of around 0.5 nm as identified for similar conditions in previous works.^19,20,22,23^ According to reports on MWL, these thin solid-like MWLs are not smooth even layers but rather noncontinuous depositions of solid-like molecular water on the hydrophilic sites. Since the presence of liquid-like MWL dramatically affects the freezing propagation rates in modes III and IV, the presence of a more or less continuous solid-like MWL arguably influences the observed freezing propagation rates in modes I and II as well.

**Figure 9:**
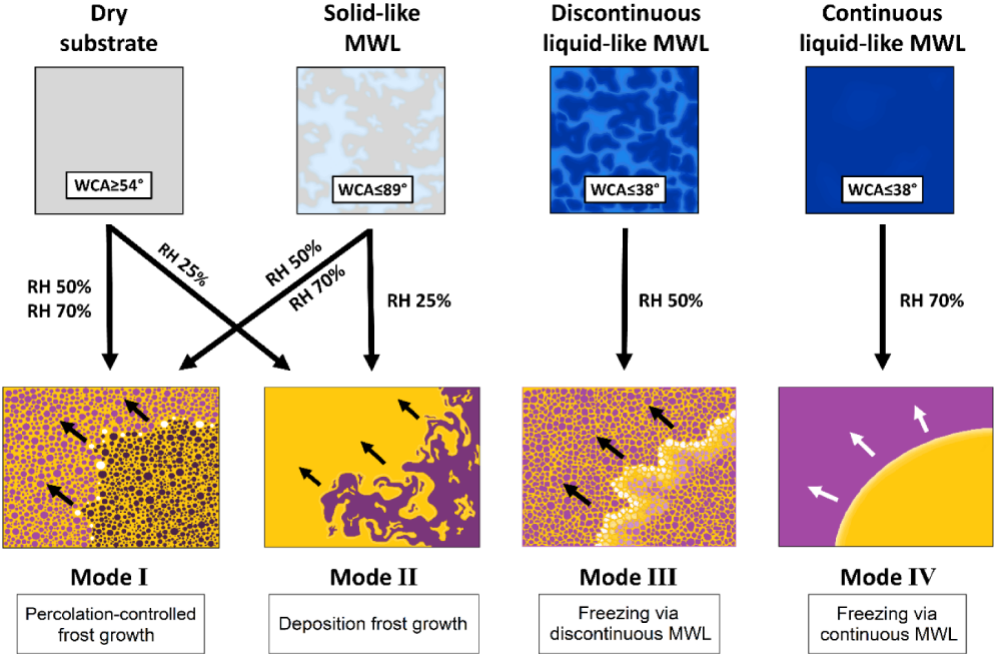
Overview of the relationship between relative humidity, surface energy, the resulting molecular water layer state, and the freezing propagation mode.

Figure 9 summarizes the proposed relation between the presence and state of molecular water layers (MWL) and the freezing mechanisms, onset times, and propagation rates observed in this work. At RH 25%, both hydrophilic and hydrophobic surfaces exhibit deposition–controlled frost, although the presence of solid-like MWL on the hydrophilic samples promotes faster freezing propagation rates. Similarly, at RH 50% and 70%, all surfaces with WCA ≥ 54° froze via percolation-limited frost propagation, while among them the hydrophilic samples with solid-like molecular water showed ten times faster freezing propagation rates. On the two most hydrophilic samples (WCA ≤ 38°) at RH 50% and 70%, the freezing events propagate via a discontinuous or continuous layer of supercooled liquid-like water, respectively.

## 4 Conclusions

In this work, the role of the molecular water layer (MWL) on the frost propagation rate and propagation front mode has been identified and studied using high-resolution thermal imaging. To do so, glass slides were functionalized to obtain a broad range of hydrophilic and hydrophobic surfaces. Exposure to selected relative humidities (RHs) and in situ monitoring of freezing at −20 °C in the presence and absence of a predeposited water droplet were monitored using infrared imaging. The systematic results obtained, and previously reported literature, allow establishing for the first time a direct relation between MWL and freezing kinetics (ranging from 0.01 to 500 mm s^–1^) and mode (from fractal to continuous front line). Depending on the MWL state, four frost propagation modes are described: ice-bridge percolation-controlled (in the presence of solid-like MWL), deposition-controlled (in the presence of solid-like continuous MWL), and continuous and discontinuous supercooled water layer-controlled freezing (in the presence of liquid-like MWL). The observations confirm that freezing in porous superhydrophobic surfaces can occur due to the presence of surface MWLs even at low RHs. The results here reported bring new insights into surface freezing and establish guidelines for the development of novel ice-controlling passive strategies using surface energy local variations.

## Supporting Information Available

Additional experimental and data analysis details, including time snapshots from thermal videos showing a freezing front propagating on all of the samples in all tested RHs (PDF)
Thermal video of percolation-controlled frost growth on sample G in 70% RH (MP4)
Thermal video of deposition frost propagation on sample A in 25% RH (MP4)
Thermal video of MWL-promoted frost propagation on sample A in 50% RH (MP4)
Thermal video of MWL-promoted frost propagation on sample A in 70% RH (MP4)

## References

[ref1] Gent R. W.; Dart N. P.; Cansdale J. T. Aircraft Icing. Philos. Trans. R. Soc. London Ser., A 2000, 358, 2873 10.1098/rsta.2000.0689.

[ref2] Cao Y.; Tan W.; Wu Z. Aircraft icing: An ongoing threat to aviation safety. Aerosp. Sci. Technol. 2018, 75, 353 10.1016/j.ast.2017.12.028.

[ref3] Laforte J. L.; Allaire M. A.; Laflamme J. State-of-the-art on power line de-icing. Atmos. Res. 1998, 46, 143 10.1016/S0169-8095(97)00057-4.

[ref4] Dehghani-Sanij A. R.; Dehghani S. R.; Naterer G. F.; Muzychka Y. S. Marine icing phenomena on vessels and offshore structures: Prediction and Analysis. Ocean Eng. 2017, 143, 1 10.1016/j.oceaneng.2017.07.049.

[ref5] Yin Z.; Yuan F.; Zhou D.; Xue M.; Luo Y.; Hong Z.; Xie C. Ultra dynamic water repellency and anti-icing performance of superhydrophobic ZnO surface on the printed circuit board (PCB). Chem. Phys. Lett. 2021, 771, 138558 10.1016/j.cplett.2021.138558.

[ref6] Tarhan C.; Çil M. A. The use of wind turbines and the problem of icing. Wind Eng. 2021, 45, 1680 10.1177/0309524X21998270.

[ref7] Wei K.; Yang Y.; Zuo H.; Zhong D. A review on ice detection technology and ice elimination technology for wind turbine. Wind Energy 2020, 23, 433 10.1002/we.2427.

[ref8] Parent O.; Ilinca A. Anti-icing and de-icing techniques for wind turbines: Critical review. Cold Reg. Sci. Technol. 2011, 65, 88 10.1016/j.coldregions.2010.01.005.

[ref9] Lin Y.; Chen H.; Wang G.; Liu A. Recent Progress in Preparation and Anti-Icing Applications of Superhydrophobic Coatings. Coatings 2018, 8, 208 10.3390/coatings8060208.

[ref10] Wang Y.; Xu Y.; Su F. Damage accumulation model of ice detach behavior in ultrasonic deicing technology. Renew. Energy 2020, 153, 1396 10.1016/j.renene.2020.02.069.

[ref11] Shi Z.; Zhao Y.; Ma C.; Zhang J. Parametric Study of Ultrasonic De-Icing Method on a Plate with Coating. Coatings 2020, 10, 631 10.3390/coatings10070631.

[ref12] Freeman A. I.; Surridge B. W. J.; Matthews M.; Stewart M.; Haygarth P. M. Understanding and managing de-icer contamination of airport surface waters: A synthesis and future perspectives. Environ. Technol. Innov. 2015, 3, 46 10.1016/j.eti.2015.01.001.

[ref13] Shen Y.; Wu X.; Tao J.; Zhu C.; Lai Y.; Chen Z. Icephobic materials: Fundamentals, performance evaluation, and applications. Prog. Mater. Sci. 2019, 103, 509 10.1016/j.pmatsci.2019.03.004.

[ref14] Brassard J. D.; Laforte C.; Guérin F.; Blackburn C. Icephobicity: Definition and Measurement Regarding Atmospheric Icing. Contam. Mitigating Polym. Coat. Extreme Environ. 2018, 284, 123 10.1007/12_2017_36.

[ref15] Strobl T.; Storm S.; Thompson D.; Hornung M.; Thielecke F. Feasibility Study of a Hybrid Ice Protection System. J. Aircr. 2015, 52, 2064 10.2514/1.C033161.

[ref16] Fortin G.; Adomou M.; Perron J. Experimental Study of Hybrid Anti-Icing Systems Combining Thermoelectric and Hydrophobic Coatings. SAE Int. J. Aerosp. 2011, 38, 3 10.4271/2011-38-0003.

[ref17] Huang X.; Tepylo N.; Pommier-Budinger V.; Budinger M.; Bonaccurso E.; Villedieu P. A survey of icephobic coatings and their potential use in a hybrid coating/active ice protection system for aerospace applications. Prog. Aeronaut. Sci. 2019, 105, 74 10.1016/j.paerosci.2019.01.002.

[ref18] Jamil M. I.; Ali A.; Haq F.; Zhang Q.; Zhan X.; Chen F. Icephobic Strategies and Materials with Superwettability: Design Principles and Mechanism. Langmuir 2018, 34, 15425 10.1021/acs.langmuir.8b03276. 30445813

[ref19] Hu J.; Xiao X. D.; Ogletree D. F.; Salmeron M. Imaging the Condensation and Evaporation of Molecularly Thin Films of Water with Nanometer Resolution. Science 1995, 268, 267 10.1126/science.268.5208.267. 17814789

[ref20] James M.; Darwish T. A.; Ciampi S.; Sylvester S. O.; Zhang Z.; Ng A.; Gooding J. J.; Hanleya L. Nanoscale condensation of water on self-assembled monolayers. Soft Matter. 2011, 7, 5309 10.1039/c1sm05096f. 21780835

[ref21] Shimizu T. K.; Maier S.; Verdaguer A.; Velasco-Velez J. J.; Salmeron M. Water at surfaces and interfaces: From molecules to ice and bulk liquid. Prog. Surf. Sci. 2018, 93, 87 10.1016/j.progsurf.2018.09.004.

[ref22] Spagnoli C.; Loos K.; Ulman A.; Cowman M. K. Imaging Structured Water and Bound Polysaccharide on Mica Surface at Ambient Temperature. J. Am. Chem. Soc. 2003, 125, 7124 10.1021/ja029721j. 12783566

[ref23] Sumner A. L.; Menke E.; Dubowski Y.; Newberg J.; Penner R.; Hemminger C.; Wingen L.; Brauers T.; Finlayson-Pitts B. The nature of water on surfaces of laboratory systems and implications for heterogeneous chemistry in the troposphere. Phys. Chem. Chem. Phys. 2004, 6, 604 10.1039/b308125g.

[ref24] Asay D. B.; Kim S. H. Evolution of the Adsorbed Water Layer Structure on Silicon Oxide at Room Temperature. J. Phys. Chem. B 2005, 109, 16760 10.1021/jp053042o. 16853134

[ref25] Bluhm H.; Inoue T.; Salmeron M. Formation of dipole-oriented water films on mica substrates at ambient conditions. Surf. Sci. 2000, 462 (1–3), L599–L602. 10.1016/S0039-6028(00)00595-1.

[ref26] Miranda P. B.; Xu L.; Shen Y. R.; Salmeron M. Icelike water monolayer adsorbed on mica at room temperature. Phys. Rev. Lett. 1998, 81 (26), 5876–5879. 10.1103/PhysRevLett.81.5876.

[ref27] Dong J.; Wang A.; Ng K. Y. S.; Mao G. Self-assembly of octadecyltrichlorosilane monolayers on silicon-based substrates by chemical vapor deposition. Thin Solid Films 2006, 515 (4), 2116–2122. 10.1016/j.tsf.2006.07.041.

[ref28] O’Brien F. E. M. The Control of Humidity by Saturated Salt Solutions. J. Sci. Instrum. 1948, 25, 73 10.1088/0950-7671/25/3/305.

[ref29] Young J. Humidity Control in the Laboratory Using Salt Solutions-A Review. Adv. J. Chem. 1967, 17, 241 10.1002/jctb.5010170901.

[ref30] Han Y.; Mayer D.; Offenhäusser A.; Ingebrandt S. Surface activation of thin silicon oxides by wet cleaning and silanization. Thin Solid Films 2006, 510, 175 10.1016/j.tsf.2005.11.048.

[ref31] Erbil H. Y. Practical Applications of Superhydrophobic Materials and Coatings: Problems and Perspectives. Langmuir 2020, 36, 2493 10.1021/acs.langmuir.9b03908. 32049544

[ref32] Nath S.; Boreyko J. B. On Localized Vapor Pressure Gradients Governing Condensation and Frost Phenomena. Langmuir 2016, 32, 8350 10.1021/acs.langmuir.6b01488. 27463696

[ref33] Boreyko J. B.; Hansen R. R.; Murphy K. R.; Nath S.; Retterer S. T.; Collier C. P. Controlling condensation and frost growth with chemical micropatterns. Sci. Rep. 2016, 6, 19131 10.1038/srep19131. 26796663 PMC4726256

[ref34] Guadarrama-Cetina J.; Mongruel A.; González- ViñViñAs W.; Beysens D. Percolation-induced frost formation. Europhys. Lett. 2013, 1, 101.

[ref35] Nath S.; Ahmadi F.; Boreyko J. How ice bridges the gap. Soft Matter. 2020, 16, 1156 10.1039/C9SM01968E. 31828263

[ref36] Paulovics D.; Raufaste C.; Frisch T.; Claudet C.; Celestini F. Dynamics of Frost Propagation on Breath Figures. Langmuir 2022, 38, 2972 10.1021/acs.langmuir.1c03463. 35196019

[ref37] Zhao Y.; Yang C. Frost spreading on microscale wettability/morphology patterned surfaces. Appl. Therm. Eng. 2017, 120, 121.

[ref38] Zhao Y.; Wang R.; Yang C. Interdroplet freezing wave propagation of condensation frosting on micropillar patterned superhydrophobic surfaces of varying pitches. Int. J. Heat Mass Transf. 2017, 108, 1048 10.1016/j.ijheatmasstransfer.2016.12.112.

[ref39] Zhao Y.; Yang C. Retarded condensate freezing propagation on superhydrophobic surfaces patterned with micropillars. Appl. Phys. Lett. 2016, 108, 061605 10.1063/1.4941927.

[ref40] Haque M.; Das S.; Betz A. Experimental investigation of condensation and freezing phenomena on hydrophilic and hydrophobic graphene coating. Appl. Therm. Eng. 2019, 160, 113987 10.1016/j.applthermaleng.2019.113987.

[ref41] Hauer L.; Wong W. S. Y.; Donadei V.; Hegner K. I.; Kondic L.; Vollmer D. How frost forms and grows on lubricated micro-and nanostructured surfaces. ACS Nano 2021, 15 (3), 4658–4668. 10.1021/acsnano.0c09152. 33647197 PMC7992192

[ref42] Shen Y.; Zou H.; Wang S. Condensation Frosting on Micropillar Surfaces – Effect of Microscale Roughness on Ice Propagation. Langmuir 2020, 36, 13563 10.1021/acs.langmuir.0c02353. 33146014

[ref43] Chavan S.; Park D.; Singla N.; Sokalski P.; Boyina K.; Miljkovic N. Effect of Latent Heat Released by Freezing Droplets during Frost Wave Propagation. Langmuir 2018, 34, 6636 10.1021/acs.langmuir.8b00916. 29733606

[ref44] Yang S.; Wu C.; Zhao G.; Sun J.; Yao X.; Ma X.; Wang Z. Condensation frosting and passive anti-frosting. Cell Rep. Phys. Sci. 2021, 2 (7), 100474 10.1016/j.xcrp.2021.100474.

[ref45] Castillo J. E.; Huang Y.; Pan Z.; Weibel J. A. Quantifying the pathways of latent heat dissipation during droplet freezing on cooled substrates. Int. J. Heat Mass Transfer 2021, 164, 120608 10.1016/j.ijheatmasstransfer.2020.120608.

[ref46] Meng Z.; Zhang P. Dynamic propagation of ice-water phase front in a supercooled water droplet. Int. J. Heat Mass Transfer 2020, 152, 119468 10.1016/j.ijheatmasstransfer.2020.119468.

[ref47] Schremb M.; Roisman I.; Tropea C. Different Outcomes after Inclined Impacts of Water Drops on a Cooled Surface. 13th Triennial International Conference On Liquid Atomization And Spray Systems, University of twente., 2015.

[ref48] Schremb M.; Roisman I.; Tropea C. Normal impact of supercooled water drops onto a smooth ice surface: experiments and modelling. J. Fluid Mech. 2018, 835, 1087 10.1017/jfm.2017.797.

[ref49] Jeong H.; Byun S.; Kim D. R.; Lee K. S. Frost growth mechanism and its behavior under ultra-low temperature conditions. Int. J. Heat Mass Transf. 2021, 169, 120941 10.1016/j.ijheatmasstransfer.2021.120941.

[ref50] Jung S.; Tiwari M. K.; Poulikakos D. Frost halos from supercooled water droplets. Proc. Natl. Acad. Sci. U. S. A. 2012, 109, 16073 10.1073/pnas.1206121109. 23012410 PMC3479582

[ref51] Pasieka J.; Nanua R.; Coulombe S.; Servio P. The crystallization of sub-cooled water: Measuring the front velocity and mushy zone composition via thermal imaging. Int. J. Heat Mass Transf. 2014, 77, 940 10.1016/j.ijheatmasstransfer.2014.06.009.

[ref52] Dukhin A. S.; Goetz P. J. Ultrasound for Characterizing Colloids. Stud. Interface Sci. 2002, 15, 75–99.

[ref53] Graeber G.; Dolder V.; Schutzius T. M.; Poulikakos D. Cascade freezing of supercooled water droplet collectives. ACS Nano 2018, 12 (11), 11274–11281. 10.1021/acsnano.8b05921. 30354059

[ref54] Wang T.; Chen M. Determining Interface Temperature During Rapid Freezing of Supercooled Water. J. Aircr. 2017, 55, 1 10.1063/1.4941927.

