# Supplementary material for: Role of Molecular Water Layer State on Freezing Front Propagation Rate and Mode Studied with Thermal Imaging
Source: Langmuir. 2024 Jun 14;40(25):12888–98. doi: 10.1021/acs.langmuir.4c00323 (PMC11210285; doi:10.1021/acs.langmuir.4c00323)
Supplement: Supplementary file 1 — la4c00323_si_001.pdf [file la4c00323_si_001.pdf]

Supporting Information

Role of Molecular Water Layer State on Freezing Front Propagation Rate and Mode Studied with Thermal Imaging

Miisa J. Tavaststjerna[a], Stephen J. Picken[b], Santiago J. Garcia[a]*

[a]Department of Aerospace Structures and Materials, Faculty of Aerospace Engineering, Delft University of Technology, Kluyverweg 1, 2629 HS, Delft, The Netherlands

E-mail: M.J.Tavaststjerna@tudelft.nl

[b]Department of Chemical Engineering, Faculty of Applied Sciences, Delft University of Technology, Van der Maasweg 9, 2629 HZ, Delft, The Netherlands

Keywords: frost, condensation, interfacial water, adsorbed water, surface wettability, thermal imaging, anti-icing

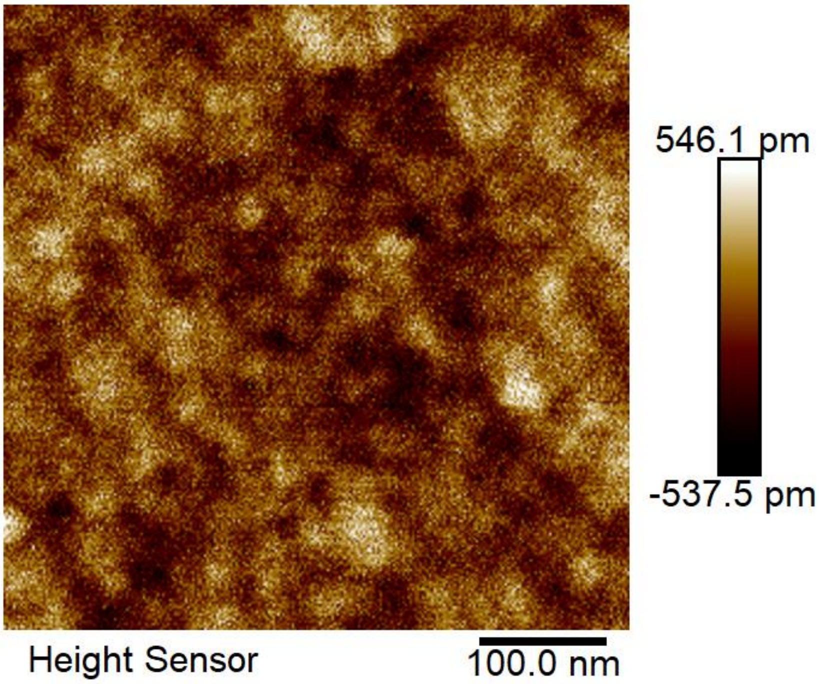

Figure S1. An AFM image of the glass slide functionalized with n-octyltrichlorosilane shows uniformity in the smooth silanized glass slides with an average roughness (R_a) of 0.125 nm.

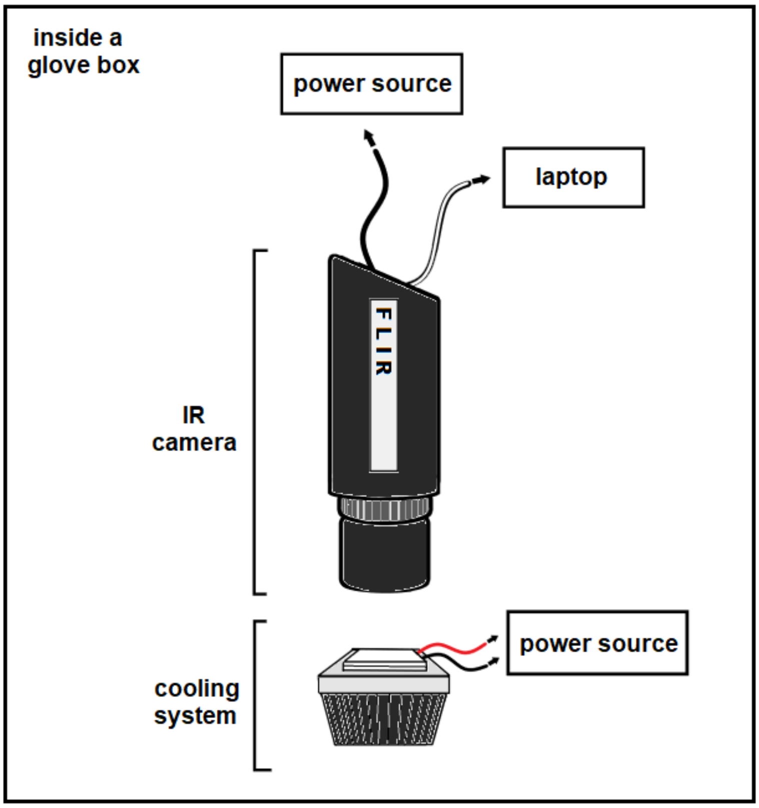

Figure S2. Schematic presentation of the experimental setup for monitoring freezing events on surfaces. Freezing events on the substrates were monitored using a FLIR A655sc thermal camera with a close-up lens (1.5 magnifying factor and 25 µm lateral resolution). The cooling system consists of two Peltier elements set in parallel (40x40 mm each) connected to a heat sink and a small fan for heat dissipation. The sample surfaces were placed on top of the two stacked Peltier plates.

Table S1. Time snapshots from IR videos* showing a freezing front propagating on the different samples exposed to **25 %** RH.

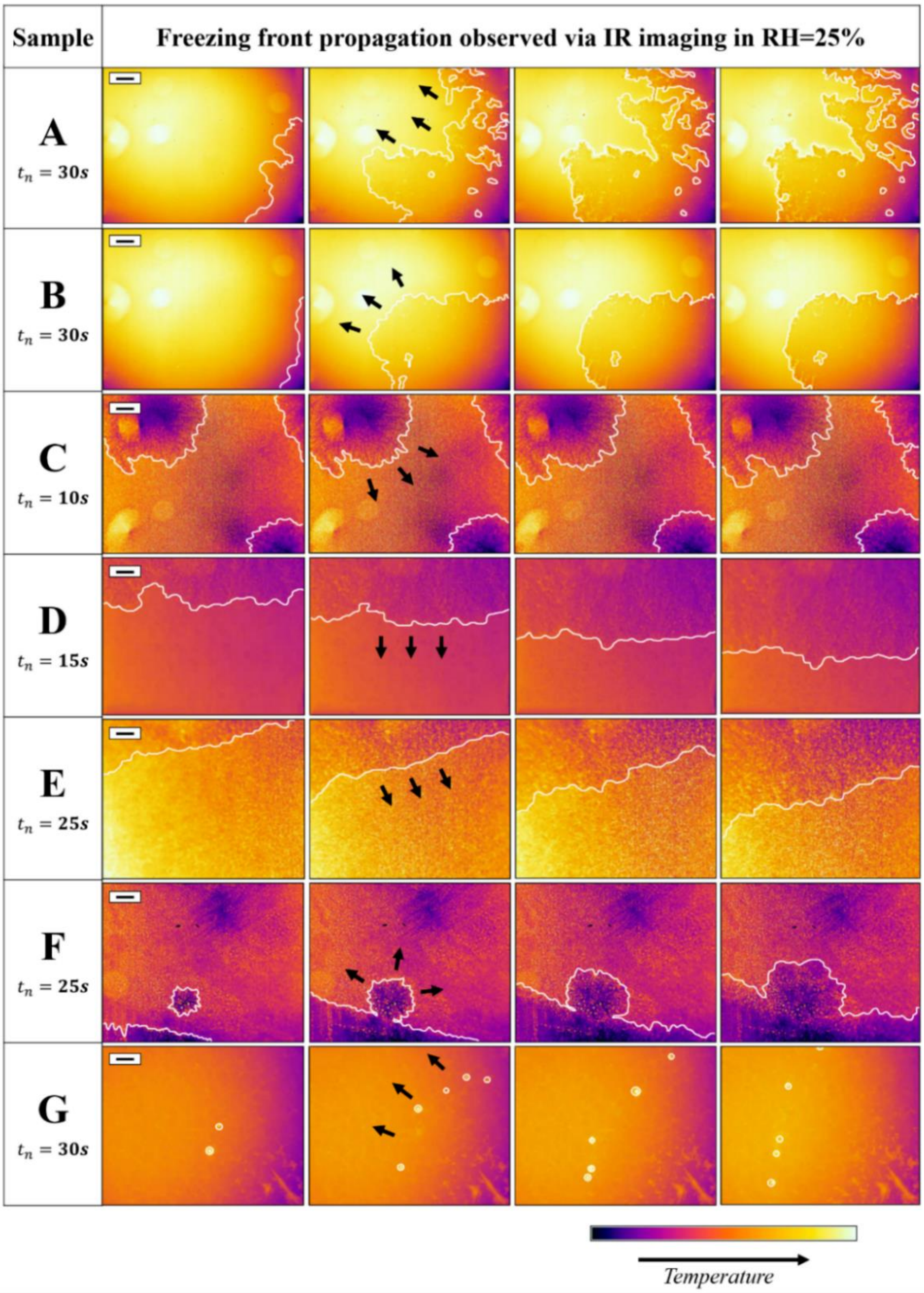

**The images are snapshots from the recorded IR imaging videos. The cooling rate in these experiments was set so that the sample surface reaches -20 °Cat 15 °Cmin^{-1}. The time step t_n between the frames is indicated separately for each sample due to variability in the frost propagation velocity. The front line of each freezing event is highlighted with a white line. Isolated freezing events are marked with white circles (sample G). The black arrows indicate the direction of the freezing front propagation. The black scale bar shown in the top corner of the first images is 1 mm. No set values were given to the temperature scale since it needed to be automatically adjusted for each video to better detect the freezing events (optimal contrast).*

Table S2. Time snapshots from IR videos showing a freezing front propagating on the different samples exposed to 50 % RH.

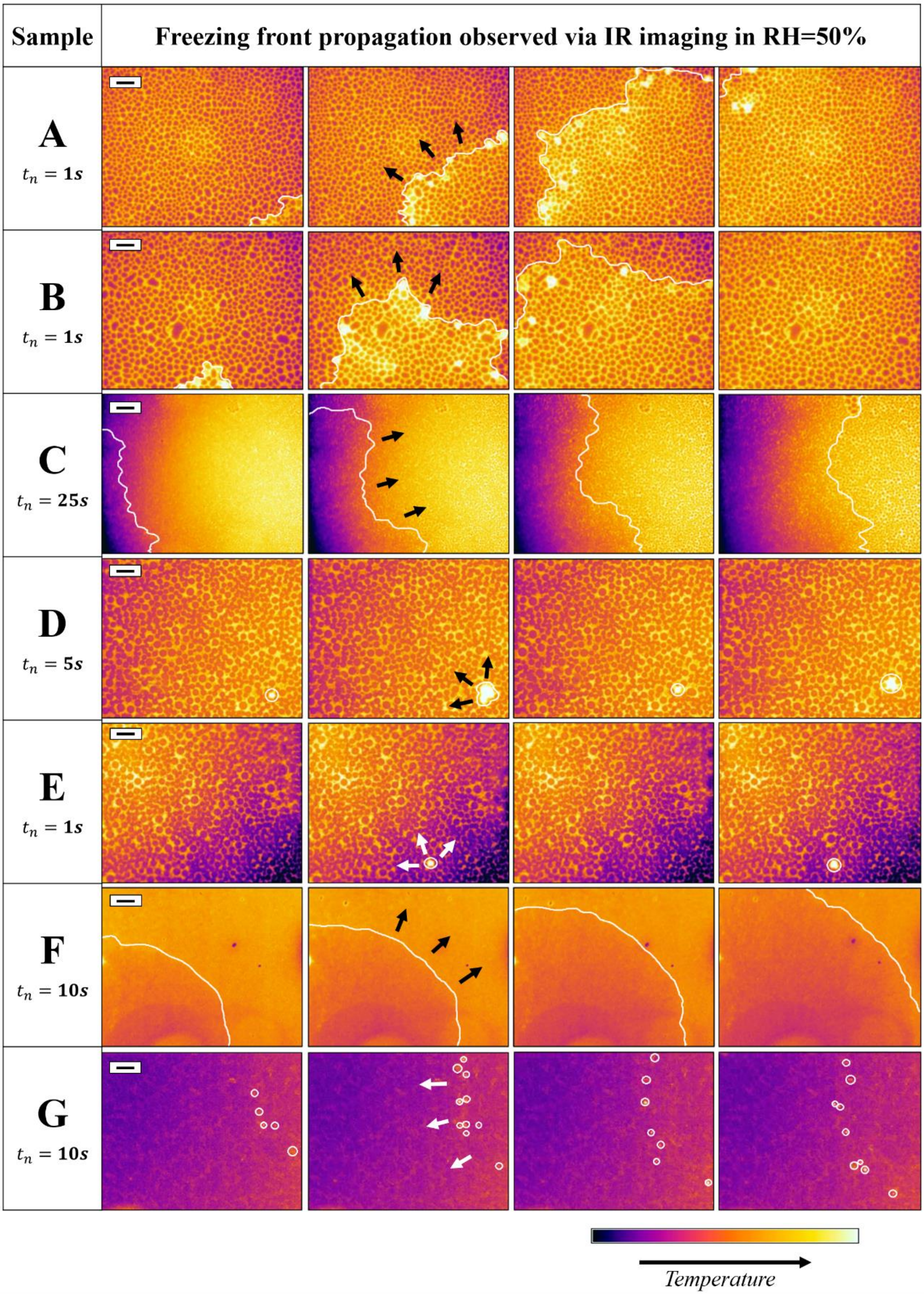

Table S3. Time snapshots from IR videos showing a freezing front propagating on the different samples exposed to **70 %** RH.

Sample	Freezing front propagation observed via IR imaging in RH=70%
**A** $t_n = \mathbf{20}\boldsymbol{ms}$	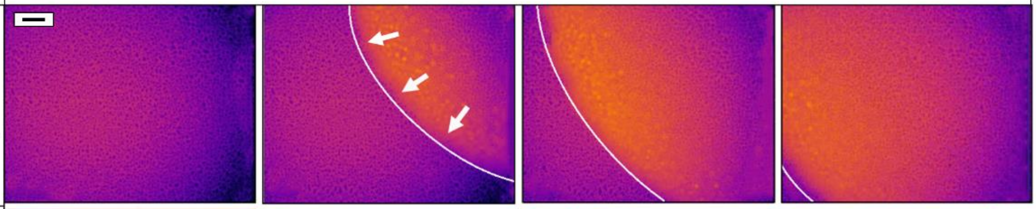
**B** $t_n = \mathbf{40}\boldsymbol{ms}$	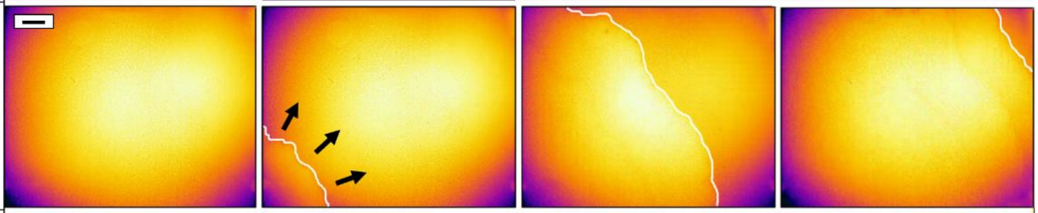
**C** $t_n = \mathbf{30}\boldsymbol{s}$	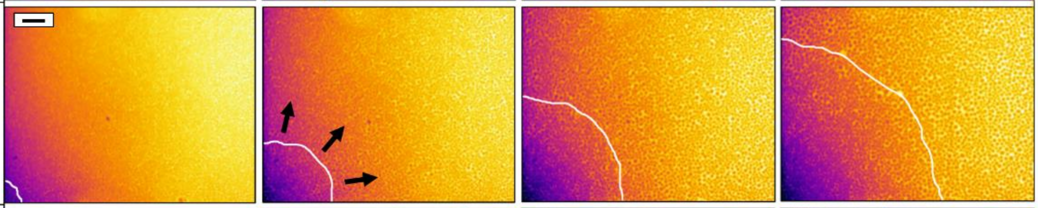
**D** $t_n = \mathbf{20}\boldsymbol{s}$	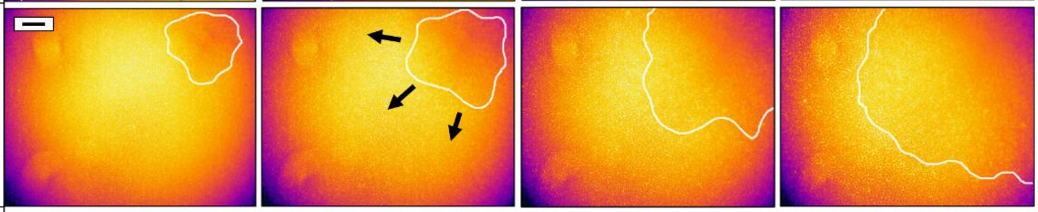
**E** $t_n = \mathbf{25}\boldsymbol{s}$	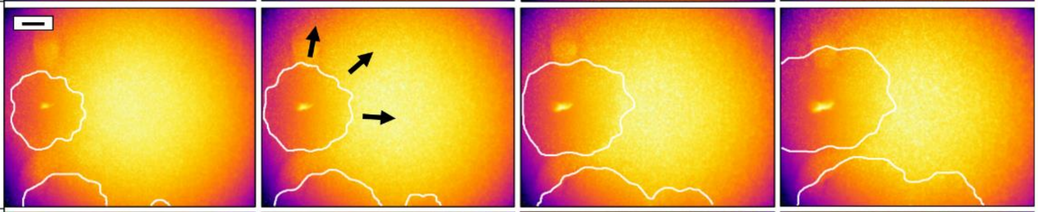
**F** $t_n = \mathbf{40}\boldsymbol{s}$	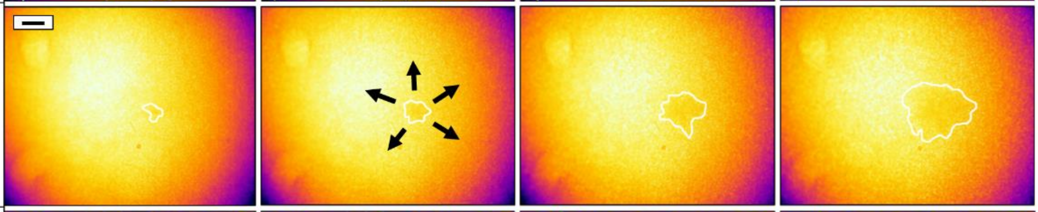
**G** $t_n = \mathbf{15}\boldsymbol{s}$	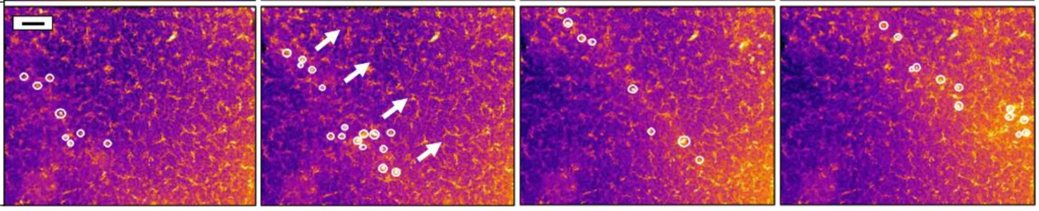

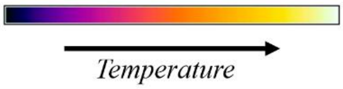

Table S4. Time snapshots from IR videos* showing a freezing front propagating on the different samples exposed to **25 %** RH in the presence of a 5 µL water droplet

Sample	Freezing front propagation observed via IR imaging in RH=25%
**A** $t_n = 30s$	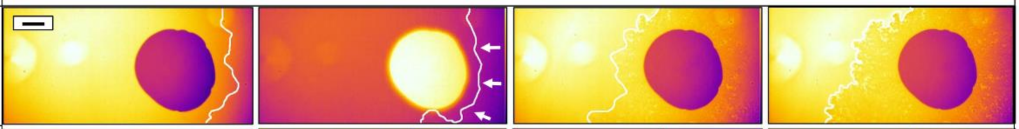
**B** $t_n = 30s$	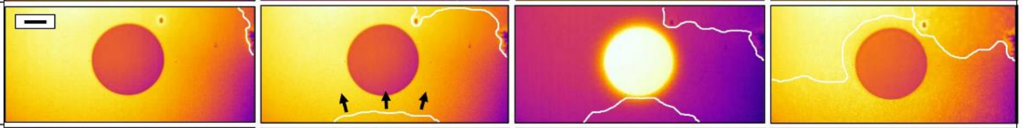
**C** $t_n = 10s$	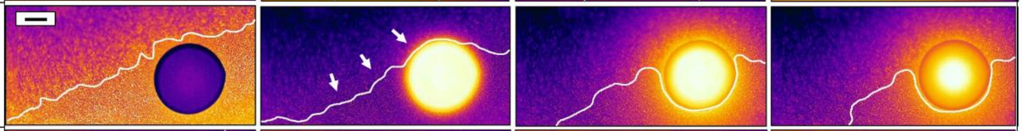
**D** $t_n = 15s$	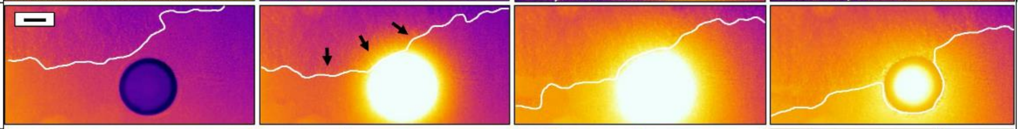
**E** $t_n = 25s$	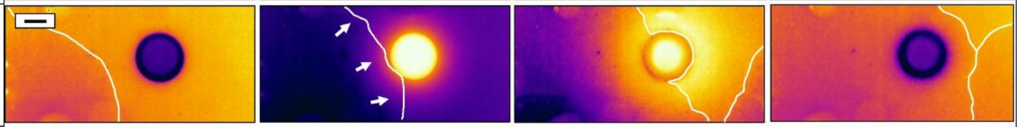
**F** $t_n = 25s$	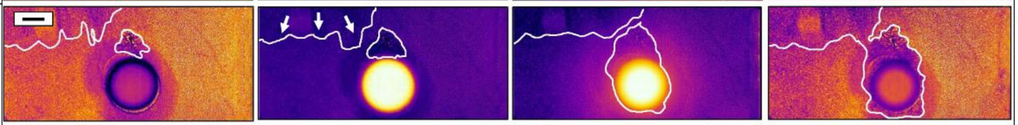
**G** $t_n = 30s$	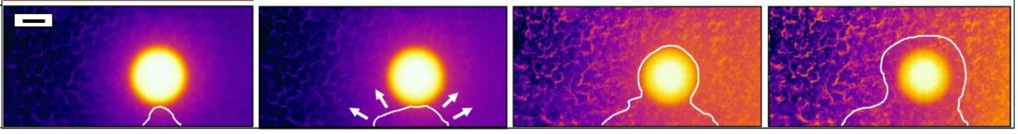

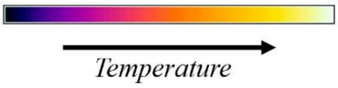

**The images were saved from a recorded IR imaging video and the time step t_n between the frames is indicated separately for each sample due to variability in the frost propagation velocity. The cooling rate in these experiments was set so that the sample surface reaches -20 ℃at 15 ℃min^{-1}. The front line of each freezing event is highlighted with a white line, and the black arrows indicate the direction of the freezing front propagation. The black scale bar shown in the top corner of the first image is 1mm. The temperature scale was automatically adjusted for each video to gain better contrast.*

Table S5. Time snapshots from IR videos showing a freezing front propagating on the different samples exposed to **50 %** RH in the presence of a 5 µL water droplet

Sample	Freezing front propagation observed via IR imaging in RH=50%
**A** $t_n = \mathbf{1s}$	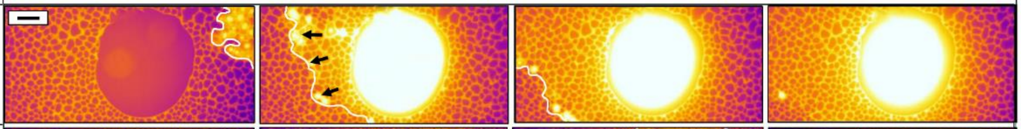
**B** $t_n = \mathbf{1s}$	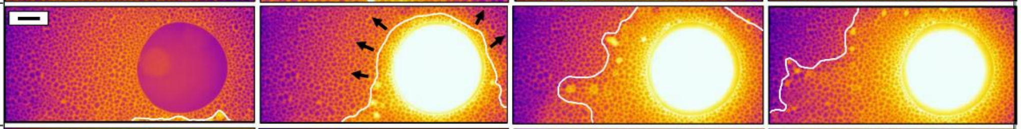
**C** $t_n = \mathbf{25s}$	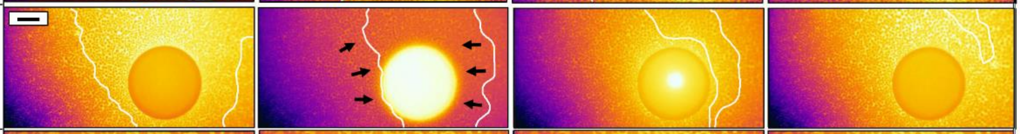
**D** $t_n = \mathbf{5s}$	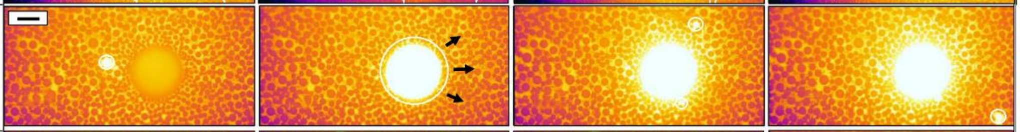
**E** $t_n = \mathbf{10s}$	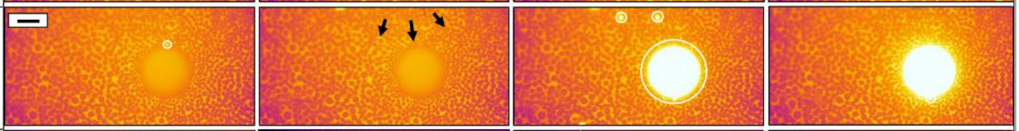
**F** $t_n = \mathbf{10s}$	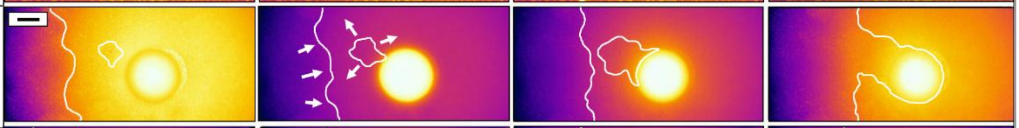
**G** $t_n = \mathbf{10s}$	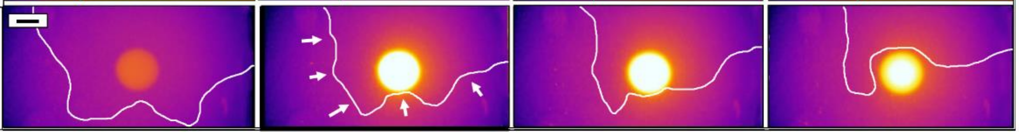

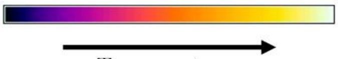

Table S6. Time snapshots from IR videos showing a freezing front propagating on the different samples exposed to **70 %** RH in the presence of a 5 µL water droplet

Sample	Freezing front propagation observed via IR imaging in RH=70%
**A** $t_n = 20ms$	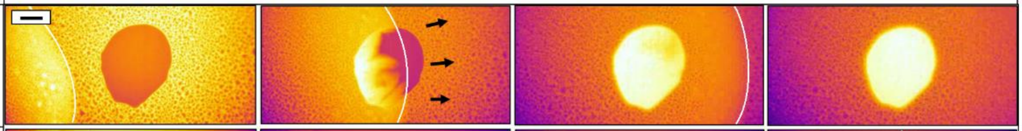
**B** $t_n = 40mss$	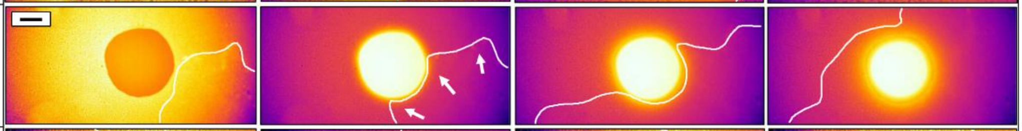
**C** $t_n = 30s$	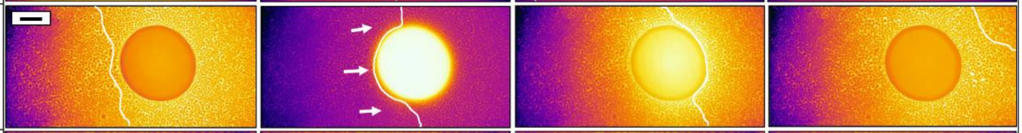
**D** $t_n = 20s$	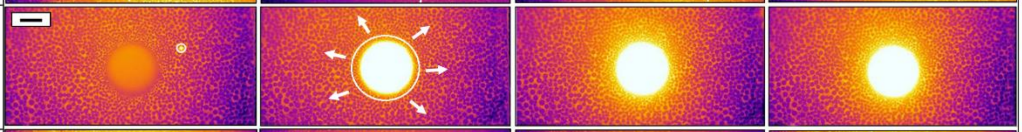
**E** $t_n = 25s$	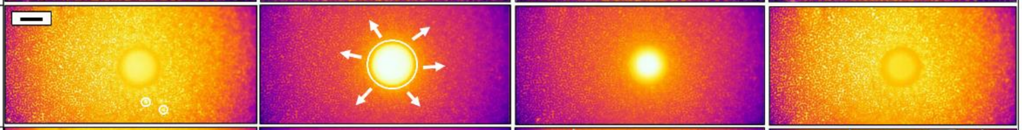
**F** $t_n = 40s$	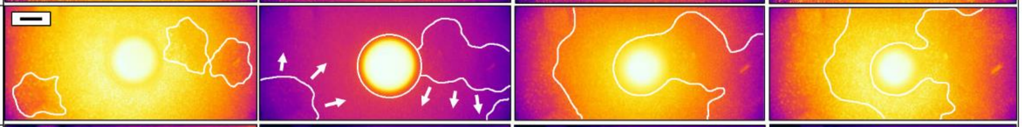
**G** $t_n = 15s$	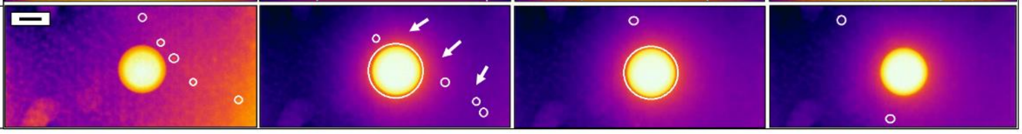

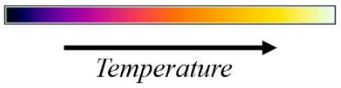

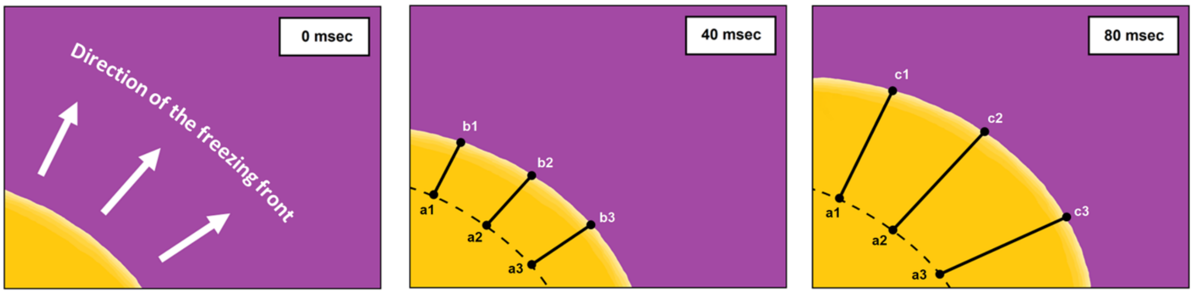

Figure S3. Calculation of the freezing front propagation rates (mm s^{-1}) from the IR video snapshots using ImageJ. The distance that the freezing front propagates between two snapshots (distance between points a-b and a-c) is divided by the time difference between the two snapshots (40 msec and 80 msec). This method is repeated for the video for a series of snapshots and points along the freezing front line to obtain an average propagation rate.

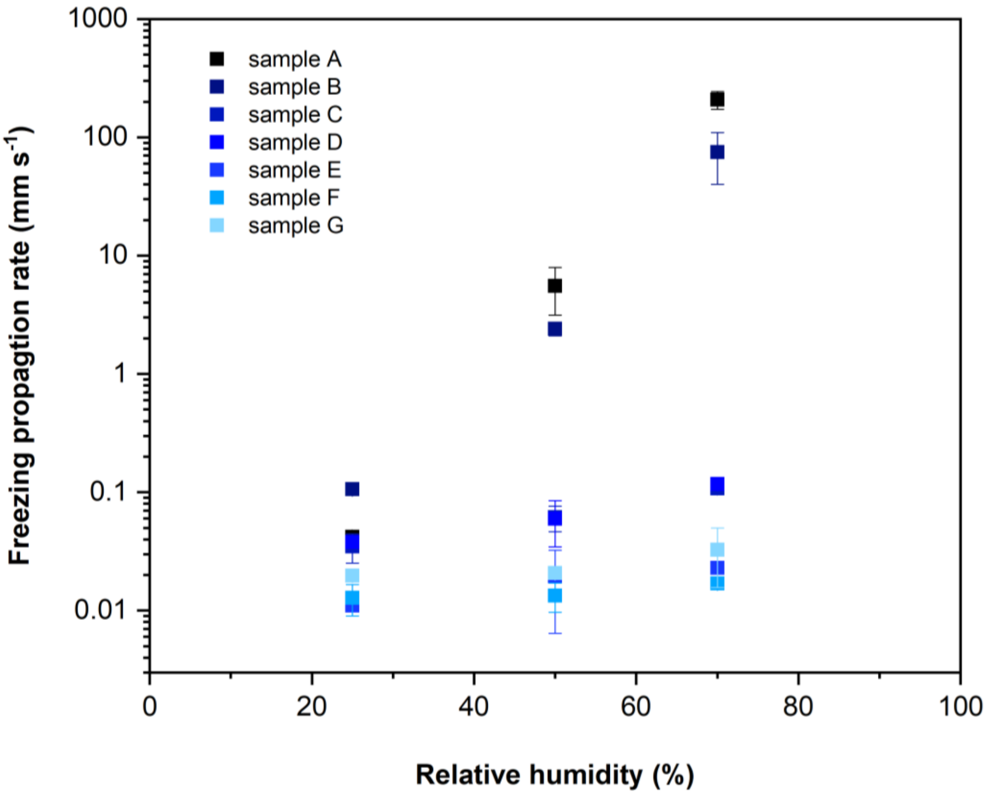

Figure S4. The measured freezing front propagation rates (mm s^{-1}) are plotted as a function of the environmental relative humidity. Note that the freezing front propagation rates are set on a logarithmic scale.

Equation to calculate SSD and nucleation pressure [32]

The critical extent of supersaturation needed for nucleation to occur on a surface can be described with the supersaturation degree (SSD) as follows:

$$SSD = \frac{p_n - p_s}{p_s} \qquad (S1)$$

Where p_n is the critical supersaturation vapor pressure required for embryo formation on the surface and p_s is the saturation vapor pressure at a given temperature. The critical supersaturation vapor pressure can be calculated from the following equation:

$$p_n = p_s \, exp\left(\frac{v}{RT_s} \sqrt{\frac{4\pi}{3} \frac{\sigma^3}{kT_s \, ln\left(\frac{I_0}{I_c}\right)} (2 + cos(\theta))(1 - cos(\theta))^2} \right) \qquad (S2)$$

Where v is the molar volume of water ($1.8 \times 10^{-5}\ m^3/mol$), R is the gas constant in $J\ /\ mol \times K$, T_s is the temperature of the surface in K, σ is the surface energy in J/m^2, k is the is the Boltzmann constant in J/K, I_0 is the kinetic constant in $m^{-2} \times s^{-1}$, I_c is the critical embryo formation rate in $m^{-2} \times s^{-1}$, and θ is the water contact angle of the surface. Here we chose $I_0 \sim 10^{29}\ m^{-2} \times s^{-1}$ and $I_c \sim 10^4\ m^{-2} \times s^{-1}$.

The saturation water vapor pressure p_s in Pa and the surface energy σ in J/m^2 at a given temperature T in K are estimated using the Goff–Gratch equation and the surface tension of liquid water or ice respect to its vapor.

For condensation:

$$p_{sc} = 10^{-7.902\left(\frac{373.15}{T}-1\right)+5.028\,\lg\left(\frac{375.15}{T}\right)-1.382\times10^{-7}\left(10^{11.344\left(\frac{1-T}{373.15}\right)}-1\right)+8.1328\times10^{-3}\left(10^{-3,491\left(\frac{373.15}{T-1}\right)}-1\right)+\lg(1013.25)} \times 100 \qquad (S3)$$

$$\sigma_c = \left(75,7 - 0,1775(T - 273.15)\right) \times 10^{-3} \qquad (S4)$$

For desublimation:

$$p_{sd} = 10^{-9.097\left(\frac{273.15}{T}-1\right)+3.57\,lg\left(\frac{273.15}{T}\right)+0.877\left(1-\frac{T}{273.15}\right)+lg(6.1173)} \times 100 \qquad (S5)$$

$$\sigma_d = \left(99.5 - 0,075(T - 273.15)\right) \times 10^{-3} \qquad (S6)$$

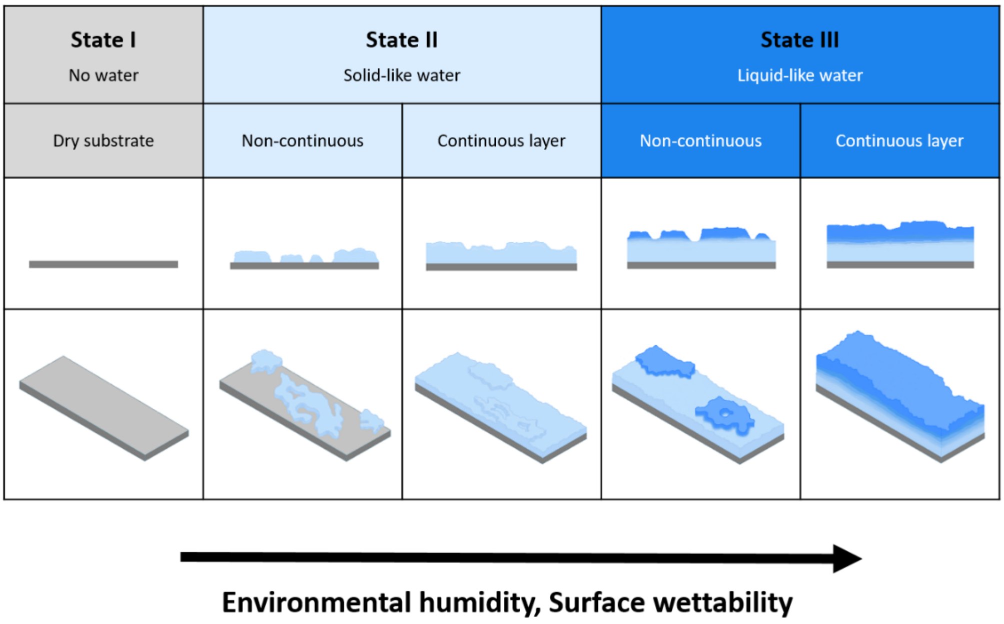

Figure S5. Illustrations of the three states of molecular water on the sample surfaces as proposed to the observed in previous research[19-24].
